# Natural biopolymers in edible coatings: Applications in food preservation

**DOI:** 10.1016/j.fochx.2025.102171

**Published:** 2025-01-15

**Authors:** Arun Karnwal, Gaurav Kumar, Rattandeep Singh, Manickam Selvaraj, Tabarak Malik, Abdel Rahman Mohammad Al Tawaha

**Affiliations:** a Department of Microbiology, Graphic Era (Deemed to be University), Dehradun 248009, Uttarakhand, India; b School of Bioengineering and Biosciences, Lovely Professional University, Punjab, India; c Department of Chemistry, Faculty of Science, King Khalid University, Abha 61413, Saudi Arabia; d Research Centre for Advanced Materials Science (RCAMS), King Khalid University, PO Box 9004, Abha 61413, Saudi Arabia; e Department of Biomedical Sciences, Institute of Health, Jimma University, Ethiopia; f Department of Biological Sciences, Al Hussein bin Talal University, PO Box 20, Maan, Jordan

**Keywords:** Edible coatings, Antioxidant, Antimicrobial, Biopolymer, Nano-emulsions, Food preservation

## Abstract

Edible coatings are revolutionizing food preservation by offering a sustainable and effective solution to key industry challenges. Made from natural biopolymers such as proteins, polysaccharides, and lipids, these coatings form a thin, edible layer on food surfaces. This barrier reduces moisture loss, protects against oxidative damage, and limits microbial growth, thereby extending shelf life while preserving food quality. Enhanced with natural additives like essential oils and antioxidants, these coatings offer antimicrobial benefits and contribute to health. Applications span from fresh produce, where they control respiration and moisture, to meat, dairy, and bakery products, maintaining sensory and nutritional properties. Innovations in coating technologies—such as composite materials, nano-emulsions, and bio-nanocomposites—are improving their mechanical strength, barrier properties, and compatibility with other preservation methods like modified atmosphere packaging. Although challenges remain in cost, consumer acceptance, and regulation, edible coatings represent a significant stride towards sustainable food systems and reduced dependence on synthetic packaging.

## Highlights

• Edible coatings extend freshness, reduce waste, and promote food sustainability.
• Plant-based coatings meet eco-friendly packaging needs in global markets.
• Nano-emulsions enhance coatings, offering quality with minimal additives.
• Antioxidant coatings preserve flavor and health, revolutionizing preservation.
• Biopolymer-based coatings combat pollution, advancing green food solutions.

## 1 Introduction

In recent years, consumer demand has shifted dramatically towards healthier and more sustainable food choices, with a clear rejection of products containing synthetic additives in favor of fresh and natural alternatives. This trend is compounded by rising environmental concerns, with a strong emphasis on minimizing plastic use and reducing food waste. At the same time, the food industry faces significant challenges in maintaining the quality and volume of perishable products from harvest to consumption. These challenges primarily stem from microbial spoilage, oxidative degradation, and sensory deterioration, which compromise food safety, pose health risks, and diminish consumer appeal (Aayush et al., 2022; Abdel Aziz & Salama, 2021; Abu-Shama et al., 2020).

**Fig. 1:**
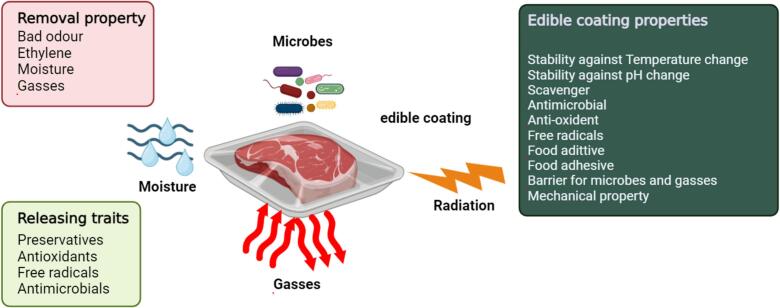
Overview of the key techno-functional properties and diverse applications of edible coatings in food preservation and enhancement.

In response to these pressures, edible coatings have emerged as a promising solution, offering an alternative to conventional plastic packaging and chemical preservatives. Edible coatings consist of thin, consumable layers applied directly to the surface of food items, preserving quality and extending shelf life (Abu-Shama et al., 2020). They are especially beneficial for perishable foods like fruits and vegetables, effectively reducing microbial contamination, oxidative damage, and moisture loss. Critical parameters for formulating edible coatings include barrier properties (such as oxygen and carbon dioxide permeability), transparency, and sensory neutrality ensuring the coatings are tasteless, odorless, and do not alter the food's appearance (Fig. 1) (Abedi et al., 2021). Despite occasional limitations, such as suboptimal mechanical properties or insufficient antimicrobial efficacy, advancements in edible coatings continue. These coatings can incorporate natural additives like antimicrobials and antioxidants, enhancing their functionality and stability. Edible coatings are biodegradable and can utilize food industry by-products, aligning with circular economy principles and contributing to sustainability by reducing food waste and reliance on plastic (Matta & Bertola, 2020). Formulations vary, ranging from hydrocolloids (polysaccharides and proteins) to lipids and composite blends, each tailored to specific food preservation needs. This adaptability, combined with cost-effectiveness and safety, positions edible coatings as a viable, environmentally-friendly packaging alternative in the modern food industry. Table 1 addresses the unique packaging requirements and problems associated with preserving the quality, safety, and shelf-life of various food categories.

**Table 1:** Overview of the main challenges and typical solutions to improve food preservation, safety, and consumer satisfaction.

**Food Type**	**Packaging Challenges**	**Packaging Issues**	**Packaging Solutions**	**Ref.**
**Fresh Produce (Fruits & Vegetables)**	- High respiration rate, - Moisture loss - Ethylene production - Mechanical damage	- Short shelf-life - Spoilage due to microbial growth - Texture loss	- Modified Atmosphere Packaging (MAP) - Biodegradable films - Cushioning materials to prevent damage	Bhat et al., 2023; Xing et al., 2016
**Meat & Poultry**	- Susceptible to microbial growth - Oxidation - Color changes	- Short shelf-life - Off-flavors due to lipid oxidation - Loss of freshness	- Vacuum Packaging - Oxygen scavengers - High-Barrier materials	da Silveira & Comettant-Rabanal, 2022; Alamdari et al., 2021a; Alamdari et al., 2021b
**Seafood**	- High perishability - Odor control - Moisture regulation	- Rapid spoilage - Texture degradation - Risk of contamination	- Active Packaging (antimicrobial coatings) - MAP - Low-Temperature packaging	Alamdari et al., 2021a; Alamdari et al., 2021b
**Dairy Products**	- Light sensitivity (UV) - Oxygen exposure - Contamination risk	- Quality loss due to oxidation - Moisture migration - Leakage	- Light-blocking materials - Barrier films (Oxygen, moisture) - Sealed containers	Daniloski et al., 2021
**Baked Goods (Bread, Pastries)**	- Staling - Moisture regulation - Mechanical damage	- Texture changes (hardening or softening) - Mold growth - Crumbling	- MAP with CO₂ control - *Re*-sealable packaging - Rigid containers to prevent damage	da Silveira & Comettant-Rabanal, 2022; Wibowo et al., 2024
**Snacks & Chips**	- Moisture sensitivity - Crushing - Oxidation	- Loss of crispness - Rancidity - Bag breakage	- High-barrier packaging (moisture, oxygen) - Nitrogen flushing - Stand-up pouches with foil layers	da Silveira & Comettant-Rabanal, 2022
**Canned & Jarred Foods**	- Corrosion of metal cans - Vacuum integrity - Glass fragility	- Risk of microbial contamination - Physical damage during transport - Shelf-life issues	- High-acid resistant cans - Hermetic sealing - Protective secondary packaging	Wibowo et al., 2024
**Frozen Foods**	- Freeze-thaw cycles - Temperature abuse - Moisture migration	- Freezer burn - Quality degradation - Packaging brittleness at low temperatures	- Moisture barrier films - Insulating materials - Freezer-friendly laminates	Bhat et al., 2023; Pié-Amill et al., 2024
**Ready-to-Eat Meals**	- Shelf stability - Heat tolerance - Odor control	- Spoilage - Packaging distortion during reheating - Odor retention	- Dual-ovenable trays - Retort pouches - High-barrier materials to prevent spoilage	Wibowo et al., 2024
**Beverages (Juices, Milk, Soft Drinks)**	- Light and oxygen exposure - Carbonation retention - Leak-proof seals	- Loss of carbonation (in carbonated drinks) - Off-flavors due to oxygen - Cap leaks	- PET bottles with oxygen barriers - Glass bottles with UV protection - Aseptic Tetra Paks	Yu et al., 2023
**Oils & Fats**	- Light and oxygen exposure - Moisture control - Container stability	- Rancidity due to oxidation - Leakage - Flavor degradation	- Dark‑tinted glass - High-barrier laminates - Airtight containers	Bizymis et al., 2024
**Confectionery (Chocolates, Candies)**	- Temperature sensitivity - Moisture migration - Aroma retention	- Bloom (sugar or fat) - Loss of flavor - Stickiness	- Foil laminates - Composite films with moisture barriers - Temperature-resistant packaging	da Silveira & Comettant-Rabanal, 2022; Behrestaghi et al., 2020
**Pet Food**	- Odor control - High-fat content - Stability during transport	- Rancidity - Pests infestation - Packaging tears	- Multi-layer bags with odor control - Foil packaging - *Re*-sealable zippers	Bhat et al., 2023; Pié-Amill et al., 2024
**Spices & Condiments**	- Aroma retention - Moisture sensitivity - UV sensitivity	- Loss of aroma - Clumping due to moisture - Flavor degradation	- Airtight glass or plastic containers - Opaque films - Sachets with high-barrier properties	Wibowo et al., 2024
**Cereals & Grains**	- Insect infestation - Moisture sensitivity - Aroma retention	- Staleness - Loss of crunchiness - Pests entry	- Laminated packaging with moisture and gas barriers - Airtight seals - Stand-up pouches with resealability	Bhat et al., 2023
**Nuts & Dried Fruits**	- High fat content - Moisture migration - Insect infestation	- Rancidity - Staleness - Pests infestation	- Vacuum packaging - Foil or metallized films - Oxygen absorbers	Wibowo et al., 2024; Ahmad et al., 2021

Fruits and vegetables (F&Vs) are rich sources of essential nutrients like minerals, antioxidants, phytochemicals, dietary fiber, and vitamins, playing a crucial role in human health (Cloete et al., 2023; Gajendran & Rajamani, 2024). Their consumption supports immune function and reduces the risk of cardiovascular diseases and cancers, making them indispensable for a balanced diet. However, managing F&Vs along the supply chain is challenging due to their postharvest physiological activity, including respiration (CO₂ release and O₂ uptake), which continues after harvest. High water content also renders F&Vs highly perishable, leading to rapid spoilage. Effective postharvest strategies must focus on controlling factors like respiration rate, ethylene production, moisture loss, and microbial contamination to extend shelf life. F&Vs are vulnerable to infections from Gram-positive and Gram-negative bacteria, fungi, yeasts, and molds, as changes in physiological and compositional properties during transport make them ideal substrates for microbial growth (Jemilakshmi et al., 2020). Key factors influencing spoilage include pH, temperature, and water activity (aw). Fruits, with a pH below 4.5, are prone to fungal growth, while vegetables, with a pH range of 4.8 to 6.5, support fungi and bacterial growth. Maintaining storage temperatures between 0 and 5 °C is critical, as higher temperatures accelerate respiration, while lower temperatures reduce microbial proliferation. However, the risk of psychrotrophic bacteria, fungi, and chilling injuries must also be managed (Kasapoğlu et al., 2022).

Globally, food loss and waste (FLW) of F&Vs exceed 20 %, with 3–18 % occurring during processing due to human errors, poor management, and technical issues. As the global population is projected to reach 9.1 billion by 2050, extending the shelf life of F&Vs is imperative to meet demand. Traditional plastic packaging falls short, both environmentally and functionally, highlighting the need for alternatives like edible coatings (Perez-Vazquez et al., 2023; Sharma, Nakano, et al., 2024). The use of biodegradable layers can greatly enhance the shelf life of fruits and vegetables by microbial spoilage, moisture loss, and minimizing respiration, providing an environmentally sustainable alternative to plastic. This review explores the potential of edible coatings as sustainable solutions, examining production techniques, incorporating bioactive compounds, and their role in reducing food waste in the supply chain.

### 1.1 Overview of food packaging challenges

**Fig. 2:**
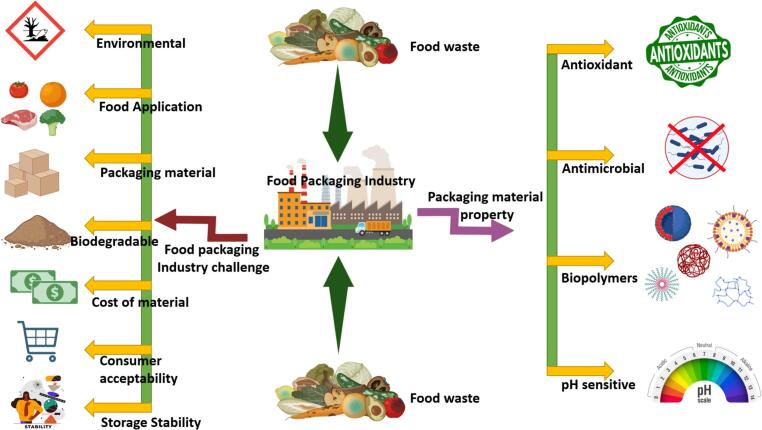
Overview of the potential applications, challenges, and recommendations for utilizing food waste and by-products in the food packaging sector.

Food packaging is pivotal in safeguarding food quality, ensuring safety, extending shelf life, and enhancing consumer convenience. However, it encounters substantial challenges related to production, environmental sustainability, consumer expectations, and regulatory compliance (Ali et al., 2024; Atta et al., 2022). Table 2 and Fig. 2 highlights the multifaceted challenges faced in food packaging, reflecting the complexity of balancing food safety, consumer expectations, environmental considerations, technological advances, and regulatory requirements.

**Table 2:** Various challenges and issues in food packaging.

**Category**	**Challenges & Issues**	**Details**	**Ref.**
**Material Selection**	Limited options for sustainable materials	Finding eco-friendly alternatives to conventional plastics that meet barrier properties and safety standards.	Atta et al., 2022; Gupta, Biswas, & Roy, 2022; Kuprina et al., 2020; Silva Cesca et al., 2024
	Compatibility of packaging materials with food types	Risk of material migration (e.g., chemicals leaching) affecting food safety and quality.	
	Availability and cost of biodegradable materials	Biodegradable options can be expensive and less readily available, especially for small-scale manufacturers.	
**Food Safety**	Contamination risks	Potential chemical, biological, or physical contaminants entering the food during packaging and storage.	Atta et al., 2022; Gupta, Biswas, & Roy, 2022; Kuprina et al., 2020; Silva Cesca et al., 2024
	Barrier properties for food preservation	Maintaining appropriate barrier properties (e.g., moisture, oxygen, light) for food shelf-life can be challenging with alternative materials.	
	Migration of harmful substances from packaging to food	Ensuring that packaging materials do not release toxins, allergens, or other harmful substances under different storage conditions.	
**Environmental Impact**	Single-use plastics	Excessive reliance on single-use plastics contributes to pollution, especially in marine environments.	Atta et al., 2022; Gupta, Biswas, & Roy, 2022; Kuprina et al., 2020; Silva Cesca et al., 2024
	Recycling limitations	Difficulty in recycling certain types of plastics and multi-layer packaging materials.	
	Carbon footprint	High carbon emissions associated with the production and transportation of traditional packaging materials like plastics and metals.	
	Biodegradability concerns	Some biodegradable materials do not decompose effectively in natural environments or regular landfills.	
**Technology & Innovation**	Limited innovation in traditional sectors	Slow adaptation of smart packaging, sensors, and other innovative solutions in traditional food sectors.	Atta et al., 2022; Gupta, Biswas, & Roy, 2022; Kuprina et al., 2020; Silva Cesca et al., 2024
	Cost of advanced packaging technology	High costs associated with smart packaging (e.g., RFID, nanotechnology) make it less accessible for small to medium enterprises (SMEs).	
	Testing and validation of new packaging technologies	Need for rigorous testing to ensure safety, quality, and functionality of new packaging innovations.	
**Regulatory Compliance**	Adherence to diverse global standards	Navigating different packaging regulations and standards across countries (e.g., EU, US FDA) for food safety and labeling.	Atta et al., 2022; Gupta, Biswas, & Roy, 2022; Kuprina et al., 2020; Silva Cesca et al., 2024
	Changing regulations	Frequent updates and changes in packaging regulations require continuous monitoring and adjustments in packaging practices.	
	Labeling requirements	Complexity in meeting labeling requirements (e.g., nutritional information, allergens) across different regions and product categories.	
**Consumer Expectations**	Demand for transparency and information	Increasing consumer demand for clear information on packaging materials, ingredients, and sustainability.	Atta et al., 2022; Gupta, Biswas, & Roy, 2022; Kuprina et al., 2020; Silva Cesca et al., 2024
	Convenience versus sustainability	Balancing convenience in packaging (e.g., single-use, resealable) with sustainable practices.	
	Food waste reduction	Ensuring that packaging minimizes food waste through improved preservation and portion sizes without compromising environmental standards.	
**Logistics & Storage**	Durability during transportation	Ensuring packaging is durable enough to withstand transport without compromising food quality and safety.	Atta et al., 2022; Gupta, Biswas, & Roy, 2022; Kuprina et al., 2020; Silva Cesca et al., 2024
	Storage stability	Packaging must maintain food stability across different temperatures and humidity levels throughout the supply chain.	
	Space efficiency	Optimizing packaging design for efficient use of storage and transportation space while minimizing material usage.	
**Cost Management**	High cost of sustainable packaging solutions	Higher costs associated with eco-friendly, reusable, or recyclable packaging alternatives compared to traditional materials.	Atta et al., 2022; Gupta, Biswas, & Roy, 2022; Kuprina et al., 2020; Silva Cesca et al., 2024
	Cost of compliance	Expenses related to compliance with international and local packaging standards, testing, and certifications.	
	Costs of technological integration	Costs of integrating technologies like QR codes, RFID, or smart sensors into packaging.	
**Supply Chain Issues**	Availability of sustainable raw materials	Fluctuations in the availability of sustainable or recycled raw materials impacting production.	Atta et al., 2022; Gupta, Biswas, & Roy, 2022; Kuprina et al., 2020; Silva Cesca et al., 2024
	Supply chain disruptions	Global disruptions (e.g., geopolitical, natural disasters) impacting material supply and costs, particularly for imported packaging components.	
	Traceability	Challenges in ensuring traceability of food products through the supply chain with traditional packaging.	
**Consumer Safety Concerns**	Presence of allergens or chemicals in packaging	Ensuring that packaging does not inadvertently introduce allergens or harmful chemicals into the food product.	Atta et al., 2022; Gupta, Biswas, & Roy, 2022; Kuprina et al., 2020; Silva Cesca et al., 2024
	Tamper-evidence and counterfeiting	Providing tamper-evident packaging and preventing counterfeiting in an increasingly global market.	
**Waste Management**	Lack of efficient disposal and recycling systems	Inadequate recycling and disposal infrastructure for food packaging waste in many regions.	Atta et al., 2022; Gupta, Biswas, & Roy, 2022; Kuprina et al., 2020; Silva Cesca et al., 2024
	Consumer behavior towards recycling	Difficulty in encouraging consistent consumer participation in recycling programs and proper disposal of packaging waste.	
	Management of post-consumer packaging waste	Handling and recycling of post-consumer waste from packaging materials, especially multi-layer and composite materials.	
**Product Integrity**	Protection of product during shelf-life	Packaging must protect against physical, chemical, and biological hazards while maintaining product freshness.	Atta et al., 2022; Gupta, Biswas, & Roy, 2022; Kuprina et al., 2020; Silva Cesca et al., 2024
	Shelf-life extension versus natural preservatives	Balancing the use of natural preservatives with the need to extend shelf life through advanced packaging solutions.	
	Preventing product tampering	Addressing security concerns to ensure that the packaging provides sufficient protection against tampering during transportation and display.

**Material selection** One critical challenge lies in **material selection**, which directly influences food preservation and shelf life. Effective packaging materials must act as a barrier to moisture, light, oxygen, and microbial contamination—factors that accelerate spoilage. For instance, packaging fresh produce like leafy greens requires breathable materials that facilitate optimal gas exchange, preserving texture and nutritional quality (Ferreira et al., 2021; Souza et al., 2020). Conversely, processed meats demand vacuum-sealed packaging to minimize oxygen exposure, thus preventing oxidation and microbial growth (Jeon et al., 2023). Selecting suitable material is not just a matter of protection; it must align with the specific preservation needs of each food type to ensure quality and minimize food waste. This complexity is compounded by the need to balance functionality with sustainability. As consumers increasingly demand eco-friendly options, finding effective and environmentally responsible materials is paramount. Inappropriate material choices can lead to compromised food-quality, shorter shelf-life, and heightened levels of food waste, impacting both the economy and the environment. Addressing these challenges requires a nuanced understanding of food properties, technological advancements in material science, and adherence to regulatory standards, ensuring that packaging solutions effectively meet safety and sustainability requirements (Abdel Aziz & Salama, 2021; Alamdari et al., 2021a; Alamdari et al., 2021b; Al-Moghazy et al., 2021).

**Environmental challenges** in food packaging are significant, mainly due to the persistent use of conventional materials like plastics, which contribute heavily to pollution and are slow to break down. Single-use plastics, prevalent in snacks, beverages, and ready-to-eat meal packaging, are significant contributors to global waste (Jeon et al., 2023; Kumar et al., 2022). Consequently, there is an increasing shift towards eco-friendly alternatives. Compostable options derived from corn starch or sugarcane are gaining traction as sustainable substitutes. However, these alternatives often fall short in durability and shelf life, leading to a challenging trade-off between environmental sustainability and practical functionality (Fitch-Vargas et al., 2024).

**Consumer demands** add another layer of complexity to packaging innovation. Modern consumers expect packaging to perform effectively and be convenient, visually appealing, and environmentally sustainable. Innovations must reconcile consumer convenience with ecological concerns by creating resealable, lightweight designs or opting for transparent materials that highlight freshness. An example is “smart” packaging, which offers real-time freshness indicators, catering to consumer desires for transparency. However, these advanced features can escalate costs and not be feasible for all manufacturers (Fernandes & Miranda, 2022; Hamed et al., 2022; Janowicz et al., 2023).

**Economic viability** is a crucial concern, as sustainable packaging solutions often have a higher price tag. Small- to medium-sized enterprises (SMEs) face particular challenges, as materials like bioplastics and smart sensors tend to be costlier. These higher production costs frequently translate to increased retail prices, potentially making eco-friendly products less competitive. Companies investing in biodegradable packaging, for instance, face material costs that are substantially higher than conventional plastics, limiting affordability for price-sensitive markets (Mahmud et al., 2024; Soro et al., 2021; Zulewska et al., 2023).

**Regulatory requirements** also play a critical role in shaping packaging innovations. Strict standards for food contact materials, enforced by regulatory bodies, restrict certain substances and necessitate extensive safety testing. For example, the European Union's stringent regulations on specific chemicals in food packaging can delay the introduction of new materials, adding to the challenges faced by manufacturers aiming to comply with evolving safety standards (Azam et al., 2022) as shown in Table 3. Table 3 provides a comparative overview of the regulations, standards, and specific restrictions governing the development and use of food edible coatings in different countries and regions. It highlights key aspects such as approval processes for ingredients, safety testing requirements, standards for biodegradable coatings, and the regulatory landscape for additives used in food coatings. The Table 3 outlines the varying regulatory approaches in the United States (FDA), European Union (EFSA), India (FSSAI), China (CFDA), and Australia (FSANZ). It emphasizes the importance of safety, environmental concerns, and functionality claims in food coating technologies.

**Table 3:** Comparative assesment between regulations, standards, and specific restrictions on the development of food edible coatings in different countries and regions (Armghan Khalid et al., 2022; Azam et al., 2022; Charlebois & Summan, 2015; Ciociola et al., 2014; European Food Safety Authority, 2011; Naqash et al., 2022; Owusu-Apenten & Vieira, 2022; Poonia & Mishra, 2022; Shukla et al., 2014; Tan et al., 2015).

**Aspect**	**United States (FDA)**	**European Union (EFSA)**	**India (FSSAI)**	**China (CFDA)**	**Australia (FSANZ)**
**Regulations on Edible Coatings**	Food coatings are regulated under 21 CFR Part 172 (Food Additives Permitted for Direct Addition to Food)	EFSA evaluates food additives for safety through the European Commission Regulation No. 1333/2008	Food coatings must comply with FSSAI's Food Safety and Standards Act, 2006 and Regulations, 2011	CFDA regulates food additives, including edible coatings, under the Food Safety Law of China	FSANZ oversees food additives under the Food Standards Code, Standard 1.3.1
**Approval of Ingredients**	Approval required for each ingredient used in food coatings	Only approved substances can be used, with specific lists of permitted additives	Only approved substances are allowed under FSSAI guidelines	Must comply with CFDA's approval process for new food additives	FSANZ requires pre-market approval for food additives
**Standards for Biodegradability**	No specific requirement for biodegradability; however, environmental concerns are encouraged	Environmental standards are part of packaging legislation; biodegradable options are encouraged	Biodegradable options are encouraged, but no specific regulation for coatings	Biodegradable and environmentally friendly coatings are promoted	Biodegradable packaging is encouraged under environmental guidelines
**Maximum Concentration of Additives**	Concentration limits vary based on specific substances (FDA guidelines for each ingredient)	EFSA provides specific concentration limits for each additive used in food coatings	Limits are outlined for each additive in FSSAI regulations	Specific limits outlined for food additives, including coatings	FSANZ sets concentration limits for each approved food additive
**Safety Testing Requirements**	Extensive toxicological testing for all ingredients in contact with food	EFSA evaluates safety data through a risk assessment process	FSSAI requires safety data for food additives used in coatings	CFDA mandates comprehensive safety tests for food additives	FSANZ mandates safety testing for all food ingredients used in coatings
**Use of Natural Ingredients**	Natural ingredients like plant-based coatings are allowed if proven safe	Natural ingredients are allowed if they meet safety standards	Plant-based and natural coatings are encouraged but must meet safety standards	Natural coatings and ingredients must meet CFDA regulations	Natural coatings are encouraged and must comply with safety regulations
**Labeling Requirements**	Coatings must be labeled with ingredients and their intended use	All ingredients, including coatings, must be clearly labeled	Ingredients of edible coatings must be listed on the label as per FSSAI regulations	All additives in coatings must be disclosed under CFDA's labeling rules	Labels must include information on edible coatings and their ingredients
**Prohibited Ingredients**	Some natural substances may be restricted due to safety concerns (e.g., certain essential oils)	Substances such as certain synthetic additives may be prohibited in coatings	Specific additives are restricted under FSSAI, such as some synthetic preservatives	Some synthetic chemicals are prohibited in food coatings	Some additives are restricted or prohibited in coatings, depending on safety assessments
**Regulations on Functional Claims**	Claims about the functionality (e.g., antimicrobial properties) of coatings require scientific substantiation	Claims related to health benefits or functionality must be substantiated by EFSA	Health and functionality claims must be supported by FSSAI-approved scientific evidence	Functional claims related to coatings require approval by CFDA	Claims about health or functionality must be substantiated under FSANZ guidelines
**Specific Restrictions on Industry Development**	Stringent approval processes for new additives and slow adaptation to novel materials	Strict EU standards can slow innovation; challenges with approval of new materials	FSSAI's slow regulatory updates may hinder the rapid adoption of new technologies	Regulatory delays and strict safety assessments can slow industry growth	Industry development is influenced by stringent regulatory reviews and slow approval processes for new additives

### 1.2 Role of edible coatings in food preservation

**Fig. 3:**
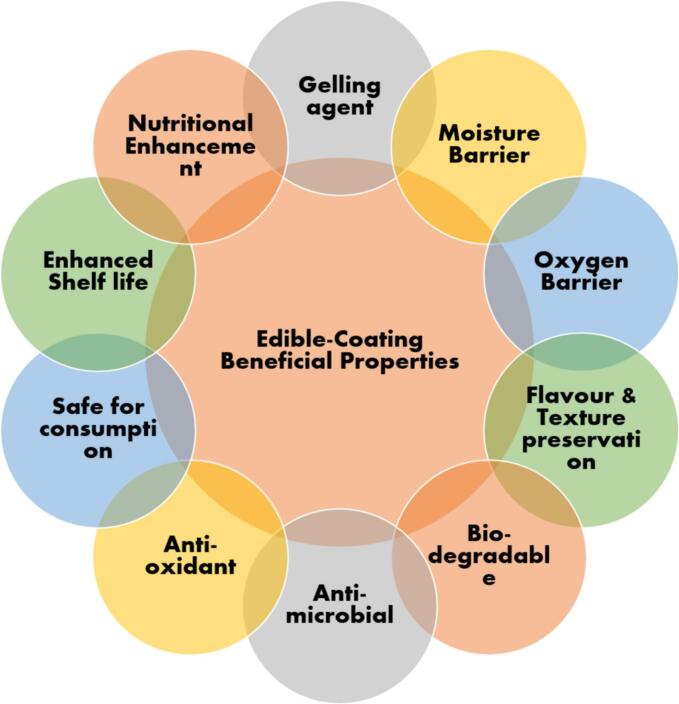
Key Properties and Benefits of Edible Coatings in Food Preservation and Quality Enhancement.

Edible coatings are advanced, thin layers of consumable materials applied to food surfaces, serving as effective hurdles towards loss of moisture, oxygen infiltration, and microbial spoilage. These coatings play a crucial role in food preservation, extending shelf life, enhancing quality, and reducing spoilage in a range of perishable products (Fig. 3) (Abdelshafy et al., 2023; Al-Ismail et al., 2020). Edible coatings, composed of natural ingredients such as lipids, polysaccharides, proteins, and lipids, are biodegradable, safe for consumption, and align with the growing demand for sustainable food solutions. An essential benefit of edible coatings is their ability to control moisture loss, which is a major factor contributing to degradation in fruits and vegetables (Ashrafi et al., 2024). During storage, moisture loss leads to weight reduction, texture deterioration, and shriveling. Polysaccharide based coatings, such as those from cellulose and pectin, form a semi-permeable layer that controls water evaporation, preserving freshness. For instance, pectin coatings applied to strawberries minimize water loss, maintain firmness, and extend shelf life significantly.

Beyond moisture management, edible coatings serve as an effective barrier against oxygen, a key trigger for oxidative reactions that can lead to off-flavors, discoloration, and the degradation of nutrients (Abdelshafy et al., 2023). Lipid based coatings, such as beeswax and carnauba wax, are particularly effective at limiting oxygen exposure (Hashemi et al., 2023). These coatings are often applied to fruits like apples, which help slow down oxidation, prevent browning, and minimize nutrient loss, thereby improving both the produce's visual appeal and market value over more extended storage periods (Table 4). Additionally, incorporating natural antimicrobials and antioxidants into edible coatings enhances their functionality, offering extended protection against spoilage and preserving food quality (Aguado et al., 2024). Essential oils, like thyme or rosemary, can be incorporated to prevent microbial growth (Pereira et al., 2019). In cheese preservation, thyme oil-infused coatings inhibit spoilage microorganisms, extending shelf life. Similarly, seafood treated with chitosan-based coatings containing natural antimicrobials shows reduced microbial spoilage and prolonged freshness (Al-Moghazy et al., 2021).

**Table 4:** Outlining the role of edible coatings in food preservation.

**Edible Coating Type**	**Macromolecule**	**Effect of Coating**	**Suitable Food Type**	**Food Example**	**Parameters of Coating**	**Application Method**	**Ref.**
**Chitosan**	Polysaccharide	Antimicrobial, moisture barrier, extends shelf life	Fruits, Vegetables, Meat, Seafood	Strawberries, Tomatoes, Chicken	Concentration (0.5 %–2 %), pH (Abdelshafy et al., 2023; Abdi et al., 2023), drying time, solvent type	Dipping, Spraying, Brushing	Kulawik et al., 2024; Reyes Méndez et al., 2023; Tran et al., 2024
**Alginate**	Polysaccharide	Reduces moisture loss, prevents lipid oxidation, controls respiration rate	Fruits, Vegetables, Fish, Meat	Apples, Carrots, Salmon	Concentration (0.5 %–3 %), Calcium chloride for gel formation	Dipping in alginate solution, Spraying	Zhu et al., 2019; Zhao et al., 20,244
**Gelatin**	Protein	Forms protective film, reduces oxygen and moisture permeability	Meat, Poultry, Dairy products	Sausages, Cheese	Concentration (1 %–5 %), drying temperature, pH (Abdi et al., 2023;Abdollahzadeh et al., 2023; Abedi et al., 2021)	Dipping, Brushing, Coating	Li et al., 2023; Reyes Méndez et al., 2023; Zhang et al., 2022
**Whey Protein**	Protein	Enhances barrier properties to oxygen, prevents browning, improves texture	Fruits, Vegetables, Dairy products	Apples, Bananas, Cheese	Concentration (3 %–10 %), pH adjustment, temperature	Dipping, Spraying	Bishnoi et al., 2022; Abedi et al., 2021
**Carboxymethyl Cellulose (CMC)**	Polysaccharide	Acts as a moisture barrier, enhances gloss, delays ripening	Fresh fruits, Bakery products	Mango, Oranges, Bread	Concentration (0.25 %–1.5 %), drying time, viscosity	Dipping, Brushing	Behrestaghi et al., 2020
**Pectin**	Polysaccharide	Forms a semi-permeable barrier, reduces dehydration and oxidative stress	Fruits, Vegetables	Lemons, Tomatoes	Concentration (1 %–3 %), pH (Abdel Aziz & Salama, 2021; Abdelshafy et al., 2023; Abdi et al., 2023), drying conditions	Dipping, Coating	Chakravartula et al., 2019; Ahmad et al., 2021
**Lipid-Based (Beeswax, Carnauba Wax)**	Lipid	Creates a hydrophobic barrier, reduces moisture loss, provides glossy finish	Fruits, Vegetables	Apples, Cucumbers	Concentration (1 %–10 %), melting temperature, drying conditions	Spraying, Dipping, Brushing	Al-Ismail et al., 2020; Devi, Das, et al., 2024; Devi, Jaiswal, & Jaiswal, 2024
**Starch-based (Corn, Potato)**	Polysaccharide	Reduces moisture migration, controls respiration, enhances food texture	Fruits, Meat, Baked goods	Strawberries, Bread	Concentration (1 %–5 %), gelatinization temperature, pH	Dipping, Coating, Spraying	Fitch-Vargas et al., 2024; Sucheta et al., 2019
**Pullulan**	Polysaccharide	Enhances barrier to oxygen and aroma compounds, provides glossy appearance	Fruits, Bakery products	Strawberries, Muffins	Concentration (2 %–6 %), drying time, plasticizer (glycerol)	Dipping, Spraying, Coating	Tang et al., 2025
**Soy Protein**	Protein	Forms a film to reduce lipid oxidation, maintains texture, slows down respiration	Fruits, Vegetables, Meat	Bell Peppers, Tomatoes, Sausage	Concentration (2 %–7 %), pH adjustment, curing conditions	Dipping, Spraying	Choeybundit et al., 2024
**Casein**	Protein	Provides a barrier to moisture and gases, improves food texture, reduces oxygen exposure	Cheese, Fruits, Baked goods	Mozzarella, Bread	Concentration (5 %–8 %), drying temperature, pH (Abdollahzadeh et al., 2023; Abedi et al., 2021)	Dipping, Coating, Brushing	Xu et al., 2023
**Gum Arabic**	Polysaccharide	Reduces moisture transfer, acts as a film-former, prevents lipid oxidation	Confectionery, Nuts, Fruits	Almonds, Candies, Citrus fruits	Concentration (3 %–6 %), viscosity, drying time	Spraying, Dipping	Hakim et al., 2023
**Xanthan Gum**	Polysaccharide	Provides barrier to moisture and gases, stabilizes food structure	Fruits, Vegetables, Meat	Strawberries, Chicken	Concentration (0.5 %–2 %), pH, drying time	Dipping, Brushing, Spraying	Gautam et al., 2024
**Composite Coatings (e.g., Chitosan + Essential Oils)**	Polysaccharide + Lipid or Protein	Enhances antimicrobial properties, improves barrier function against gases and moisture	Fruits, Vegetables, Meat	Blueberries, Lettuce, Fish fillets	Concentration of components, ratio of mix, solvent type	Dipping, Coating, Spraying	Chakravartula et al., 2019; Gautam et al., 2024
**Corn Zein**	Protein	Provides excellent barrier to oxygen, reduces moisture loss, enhances gloss	Nuts, Fruits, Baked goods	Pecans, Grapes, Crackers	Concentration (2 %–5 %), drying conditions, pH	Spraying, Dipping	Pereira et al., 2019; Almeida et al., 2024

Table 4 includes a diverse range of edible coatings, their effects on food preservation, specific food applications, and technical parameters to ensure effective preservation. It highlights the versatility of edible coatings and their contribution to extending shelf life, maintaining quality, and enhancing the appearance of food products. Edible coatings also address postharvest food waste by delaying ripening and extending the freshness of sensitive produce. Avocados, for example, benefit from coatings that slow ethylene production, curbing rapid ripening and decay during storage and transport (Funes et al., 2023). Moreover, edible coatings can carry vitamins, probiotics, and other health-boosting compounds. Yogurt-coated fruit snacks preserve the fruit and deliver added nutritional benefits, offering consumers enhanced health value (Hao et al., 2022). Edible coatings are a versatile and highly effective method of food preservation. Advanced moisture control, oxygen resistance, antimicrobial protection, and nutrient enrichment provide a sustainable solution for extending shelf life, reducing food waste, and meeting consumer expectations for natural, safe, high-quality food products.

## 2 Types, composition, characteristics, and functions of edible coatings

**Fig. 4:**
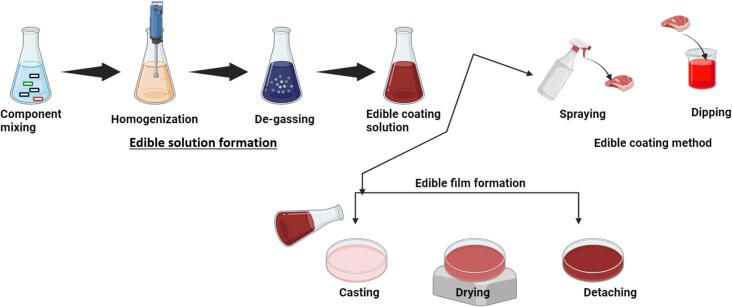
Preparation process of edible films and edible coatings.

**Fig. 5:**
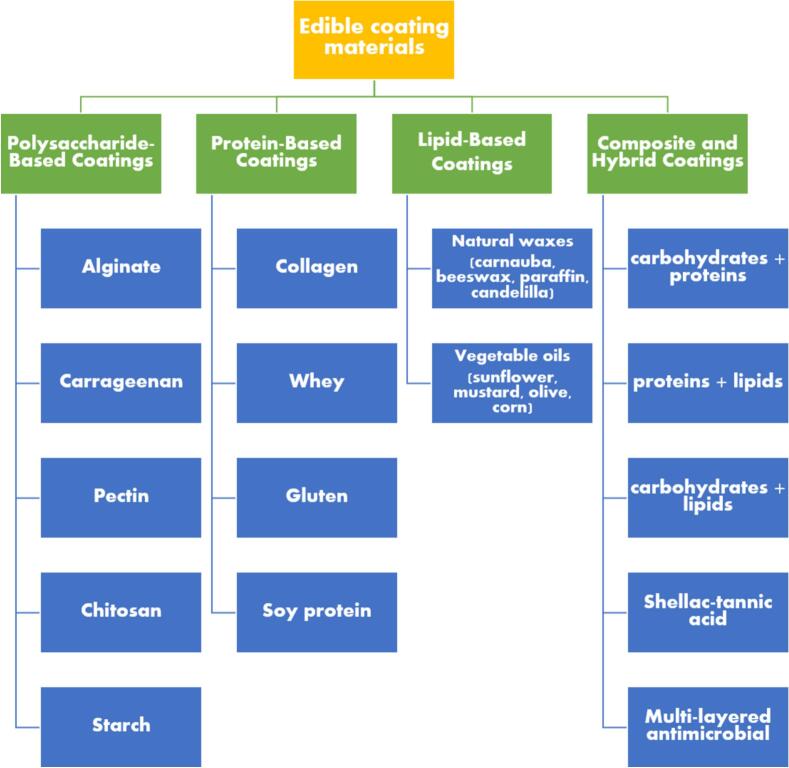
Classification of Edible Coating Materials for Enhanced Food Preservation: A comprehensive overview categorizing polysaccharide-based, protein-based, lipid-based, and composite/hybrid coatings.

Edible coatings and films from consumable ingredients offer an advanced method for maintaining food's physicochemical and sensory qualities throughout its shelf life. These coatings play crucial technical roles by enhancing visual appeal and texture while providing practical barriers against carbon dioxide, oxygen, and moisture. Edible coatings serve as delivery systems for bioactive compounds, enhancing the nutritional and functional properties of food, but they are also composed of food-grade biopolymers like lipids, proteins, polysaccharides, and their derivatives (Fig. 5) (Azeredo et al., 2022; Bizymis & Tzia, 2022; Gohargani et al., 2021). These materials are gaining popularity in the food industry as innovative and sustainable packaging options (Table 5). Typically, edible coatings are formulated as blends of films and functional additives, acting as primary packaging solutions that safeguard food safety and quality while minimizing environmental impact. An illustration is given in Fig. 4 about the methodology used for preparation of edible film and coating.

**Table 5:** Impact of Edible Coatings Containing Macro-Molecules on Shelf Life and Quality of Various Food Products (Das et al., 2020; Eshghi et al., 2022; Galus et al., 2021; Rodriguez-Garcia et al., 2016; Sucheta et al., 2019; Tabassum et al., 2018; Zam, 2019).

**Macro-Molecule**	**Food Product**	**Remarkable** **Component**	**Edible Coating**	**Improvement**	**Storage (Days)**	**Storage T (°C)**
Polysaccharide	Tomatoes	Oregano EOs	Oregano EOs + Pectin (36.1 mg/mL)	Reduced fungal deterioration and increased antioxidant efficiency	12	25
		beetroot powder/ Cornflour powder/Corn-flour starch	Beetroot powder corn-flour+ Starch +Commercial pectin	Reduced respiration rate, decreased weight loss, and decreased degradation percentage	30	25
		Aloe-vera ZnO-NPs	ZnO-NPs + *Aloe vera* +Alginate	No deterioration in storage	16	28
		Sweet-orange EOs	sweet orange EOs (5 %) + Sodium alginate	Reduced weight loss and the elimination of Salmonella and Listeria's sessile and planktonic forms	15	22
	Strawberry	Persian Gum, Low methoxyl pectin, methyl cellulose	methyl cellulose + Glycerol (1 %) Low methoxyl pectin+ Glycerol (2 %) Persian Gum+ Glycerol (4 %)	Reduced the speed of decomposition in anthocyanins, total phenolics, and ascorbic acid.	16	4
		Cinnamon EOs	cinnamon EO + Pullulan +	Delayed firmness, decay percentage, and mass loss	6	20
	Grapes	Bergamot EOs	bergamot EO + HPMC+ Chitosan	reduction in weight, decrease in respiration rate, and enhancement of firmness, Antibacterial activity.	22	2
		Gum-ghatti	Gum ghatti (1 %) + Chitosan (1 %)	Minimization of yeast-mold growth, Preserve phenolic acid content	60	1
	Red bell peppers	–	Gelatin+Chitosan	Nutritional content maintenance, Respiration rate maintenance, Microbial spoilage reduction	7	21
	Oranges	–	guar gum + Pea starch	prolonging the shelf life, Better sensory scores and a greater sense of off flavors	7	20
	Cherry fruits	Olive-leaf extract	Olive leaf extract + Chitosan (1 %) + Alginate (3 %)	Delays in maturation and an increase in anthocyanins	20	25
	Kiwifruit	Vanillin, Ascorbic acid	vanillin (1 %) + ascorbic acid (0.5 %) + Sodium alginate (2 %)	Reduced ascorbic acid loss and degradation	7	5
	Apples, Potatoes, Carrots	Trans-glutaminase	transglutaminase + pectin +Whey protein pectin	Reduced weight loss, microbial growth inhibition, and retention of antioxidant activity	10	5
	Pineapple	Cinnamon EOs	Cinnamon essential oil (0.5 %) + Chitosan (2 %) + PET	reduced weight loss, and microorganism proliferation inhibition	15	5
Protein	Fresh-cut apple	Ferulic acid	ferulic acid +Soy protein	Controlling weight loss and firmness	7	10
	Fresh-cut apple	Trehalose and Glycerol	trehalose + glycerol + Whey protein nanofibrils	Regulation of enzymatic browning	4	10
	Tomatoes	Clove oil	Clove oil + Xanthan gum+ Whey protein	Stability and coloration enhancements; inhibition of respiration	20	15
	Fresh-cut eggplant	Cysteine	cysteine (1 %) + Soy protein	Regulation of enzymatic browning	5	8
	Pears	Lemon oil	lemon oil (1 %) + Whey protein (8 %)	decrease in color changes, decrease in hardness loss, and decrease in flavonoid and polyphenol loss	28	4
	Walnuts	–	Walnut flour protein	Preserving the sensory qualities and preventing lipid degradation	84	40

**Fig. 6:**
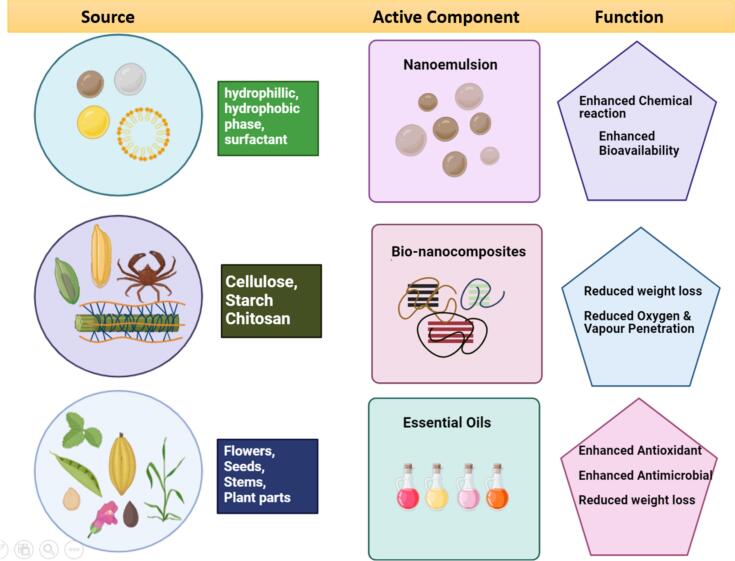
Functional Roles of Principal Active Components and their natural sources in Optimizing Edible Coatings for Food Quality and Safety.

**Fig. 7:**
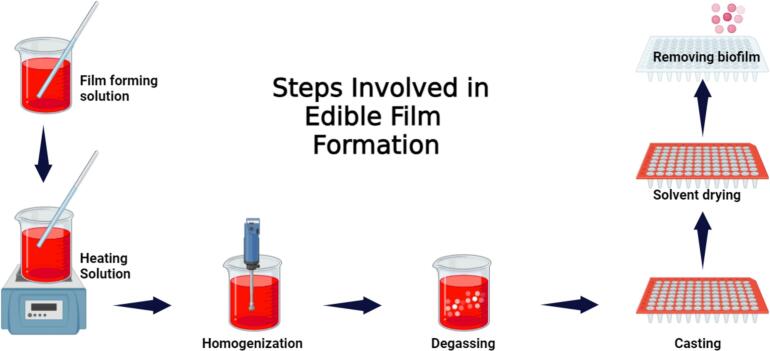
Schematic representation of the steps involved in the formation of edible films, from material preparation to final film drying.

**Fig. 8:**
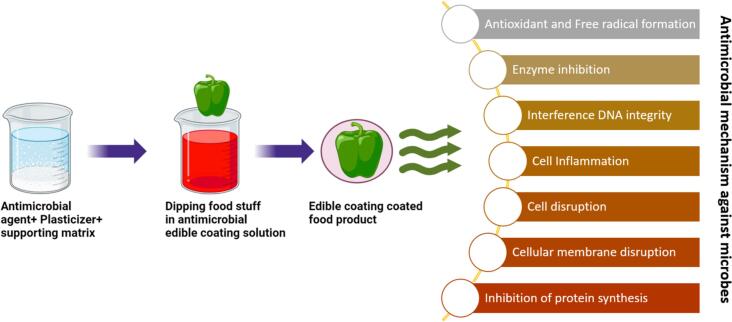
Mechanistic Insights into the Role of Antimicrobial Coatings in Food Packaging for Pathogen Control.

**Fig. 9:**
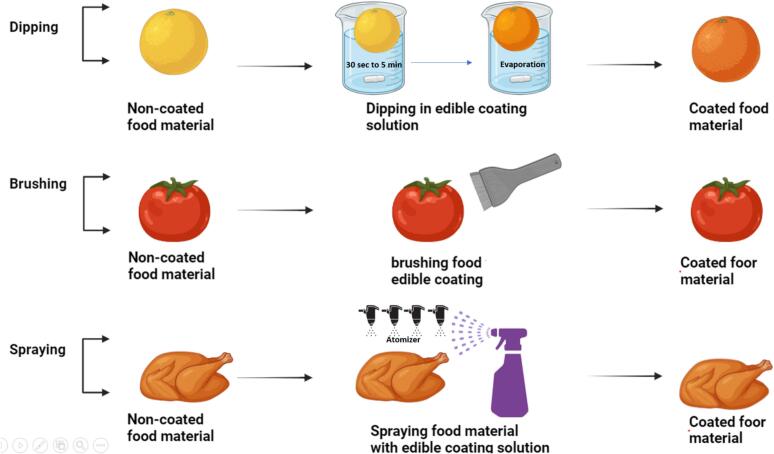
Different Methods of Applying Coating Materials (Dipping, Brushing and Spraying) to Enhance Food Product Quality and Shelf-Life.

Biopolymers play a pivotal role in developing edible coatings, with proteins and polysaccharides providing practical barriers against oxygen, lipids, and aromas, albeit with moderate mechanical strength and high water permeability (Gupta, Cherwoo, et al., 2022). Conversely, lipids are prized for their cohesive properties, offering desirable gloss and acting as an effective barrier to water loss when the transition temperature is reached (González-López et al., 2023; Janowicz et al., 2022). These biopolymers are frequently utilized in formulations to improve the physicochemical properties of edible coatings, resulting in coatings with remarkable performance. Furthermore, incorporating essential oils (EOs), bio-nanocomposites, and inorganic nanoparticles (NPs) into these mixtures significantly enhances their functionality, improving antimicrobial activity, mechanical strength, and overall stability (Ribeiro et al., 2020). This combination of biopolymers and functional additives ensures the development of cutting-edge, sustainable food packaging solutions capable of preserving food quality and extending shelf life.

### 2.1 Polysaccharide-based coatings

Since polysaccharides are inexpensive, environmentally friendly, non-allergic, and biodegradable, they quickly become the best surface coating agents. Edible coatings based on polysaccharides are extensively utilized in the food industry and commonly incorporate polymeric components such as alginate, carrageenan, pectin, chitosan, starch, or combinations of these polymers. These polysaccharides, sourced from diverse natural origins, offer distinct functional properties that enhance food preservation and safety, making them indispensable in modern food packaging solutions (Cruz-Monterrosa et al., 2023; Hashemi et al., 2023; Pillai et al., 2024).

**Chitosan**, derived from the deacetylation of chitin found in crustacean exoskeletons and fungal cell walls, is particularly noted for its antimicrobial properties. The biopolymer exerts its antimicrobial effects through electrostatic interactions between its amino groups and bacterial cell membranes, alongside its role as a metal scavenger, hindering the nutrient transport necessary for microbial growth (Abdollahzadeh et al., 2023; Adhikari et al., 2023). Chitosan demonstrates remarkable versatility through its broad range of applications. It functions effectively in antimicrobial films incorporating bioactive compounds, such as cinnamaldehyde, to preserve milk (Akhavan et al., 2021). Additionally, chitosan is a bioactive layer in paper-based packaging systems designed to inhibit yeast growth in orange juice (Demircan & Velioglu, 2024).

**Starch**, a readily available polysaccharide extracted from various agricultural sources like grains and tubers, has gained widespread application due to its excellent film-forming capabilities. Structurally, it consists of two primary components: amylose and amylopectin. The specific ratio between these fractions determines the starch's solubility and film-forming properties (Almajidi et al., 2024; Berti et al., 2020). Adding plasticizers can greatly improve the internal elasticity of starch-based films, increasing tensile strength while decreasing water solubility (Mendonça et al., 2024). This modification makes starch more versatile for diverse applications in biodegradable packaging and other industries. Starch coatings are often combined with surfactants or hydrophobic polymers to improve water resistance further, as seen in using sorbitol as a plasticizer in loquat seed starch coatings for strawberries (Costa et al., 2023).

**Pectin**, extracted from fruit cell walls, shares similar film-forming characteristics with starch, whose methoxy content is critical in its properties. As a biodegradable, FDA-approved material, pectin is often used alone or in combination with other biopolymers and essential oils to enhance its mechanical properties and solubility (Abdalla et al., 2023; Chakravartula et al., 2019). A prime example includes using pectin combined with cinnamon essential oil to extend the shelf life of fresh-cut apples, preserving their sensory qualities (Naqash et al., 2022). Innovations such as integrating cellulose nanocrystals with pectin have significantly improved barrier and antioxidant properties, enhancing food preservation by reducing water vapor and oxygen transmission rates (Wu et al., 2023; Wu, Jiang et al., 2024).

**Alginate** Edible biopolymers such as alginate, derived from the seaweed species *Phaeophyceae*, offer exceptional potential in food preservation due to their unique gelling, thickening, and film-forming properties. Alginate's exceptional efficiency originates from its unusual ability to interact with calcium ions, resulting in semi-permeable membranes (Zhu et al., 2019). These membranes are essential in maintaining the delicate balance of carbon dioxide and oxygen during respiration, vital for the sensory and visual integrity of perishable and fresh produce meals. By controlling gas exchange, alginate avoids discoloration, retains texture and improves the overall appeal of cheese, poultry, meat, vegetables, and fruits. As a result, alginate-based coatings have grown in popularity due to their potential to dramatically lengthen shelf life while keeping desirable visual and sensory attributes (Vlahova-Vangelova et al., 2022).

**Pullulan**, a linear extracellular polysaccharide made by fungus from sugar and starch, is gaining popularity quickly because of its exceptional properties, including excellent solubility, non-toxicity, and biodegradability. These attributes make it an excellent contender for sophisticated film production. Although achieving optimal flexibility remains difficult, pullulan-based films have excellent barrier characteristics against oil and vitamin oxidation. Recent advances, notably in high-throughput manufacturing processes, have revealed tremendous antibacterial potential in these films. Incorporating β-glucan into pullulan matrices improves tensile strength, water solubility, and elongation at break, leading to better functional performance of the films (Tang et al., 2025; Wani et al., 2024).

The success of edible films relies heavily on the choice of source materials, with the critical balance of hydrophobic and hydrophilic components. Natural gums such as guar, xanthan, and Arabic play essential roles in film formulations. According to research, adding more modified starch and psyllium gum can improve film qualities, particularly elongation at break (Mohajeri et al., 2023). Furthermore, mucilage, a plant-derived polymeric gel, has emerged as an interesting new element, drawing research attention for its potential to improve the mechanical and barrier properties of edible films.

### 2.2 Protein-based coatings

Proteins, known for their diverse functional properties, are essential in optimizing the rheological behavior necessary for surface coatings and facilitating effective film formation. Sourced from plant and animal origins, these biomaterials offer various technical functionalities vital for food preservation. Plant-based proteins such as gluten, soy, and whey and animal-derived proteins like gelatin and collagen provide distinct food processing and packaging advantages. The adaptability of protein-based coatings and films makes them crucial for maintaining food quality and extending shelf life (Dhumal et al., 2019).

**Collagen**, a plentiful byproduct of the meat industry derived from bones, cartilage, and skin, features an extended helical protein structure that renders it particularly effective for food packaging applications. Its use is especially beneficial in preserving meat, poultry, and seafood. Collagen-based coatings are recognized for their superior moisture and oxygen barrier properties, which help inhibit microbial growth and significantly enhance food preservation. In sausage packaging, collagen minimizes fat uptake and improves the product's texture and flow characteristics. Collagen bolsters structural integrity and reduces lipid oxidation when co-extruded with meat into sausage casings, resulting in a more stable and longer-lasting product (Cao et al., 2022; Cui et al., 2024).

Whey, a nutrient-rich byproduct of the dairy industry, is notable for its water solubility and distinctive yellow-green hue. This versatile ingredient is categorized into two main types: whey protein isolate (WPI) and whey protein concentrate (WPC) (Azizi et al., 2024). WPI is particularly prized for its higher protein content and lower fats, carbohydrates, and lactose levels. The increased protein concentration in whey facilitates the formation of robust, three-dimensional gel networks, positioning it as an excellent candidate for innovative edible packaging solutions. Compared to polysaccharides, pure whey protein films exhibit remarkable film-forming capabilities, enhanced physical strength, and superior barrier properties. Additionally, whey is rich in naturally occurring bioactive compounds—such as lysozyme, lactoperoxidase, lactoferrin, and cysteine—which provide inherent antimicrobial and antioxidant benefits (Jeon et al., 2023). For example, an edible coating derived from whey protein concentrate and infused with cinnamon extract has been shown to preserve the sensory qualities of curd cheese while effectively inhibiting the molds and yeast growth during storage (Alizadeh-Sani et al., 2019; Kandasamy et al., 2021; Mileriene et al., 2021). These examples highlight the significant potential of protein-based coatings to revolutionize food packaging, enhance product quality, extend shelf life, and promote sustainable practices.

**Gluten**, a cereal-based globular protein, stands apart from other protein-based polymers due to its insolubility in water. Composed of gliadin and glutenin, the key amino acids responsible for film formation, gluten-based edible coatings demonstrate superior barrier properties against oxygen, carbon dioxide, and hydrophobic substances (Panthi et al., 2024). However, gluten's inherent drawbacks include its limited elongation, structural rigidity, and water vapor barrier properties. Additionally, gluten is a known allergen, which poses a significant challenge in its application, particularly for consumers with gluten intolerance, as it can trigger celiac disease. Despite these concerns, no reports have emerged indicating that gluten-based edible films cause allergic reactions, and using gluten below allergenic thresholds presents a promising avenue for broadening market acceptance (Ananey-Obiri et al., 2020; Xu et al., 2023). When applied to fruits and vegetables, gluten-based coatings create a fibrous matrix that enhances product resilience and aesthetic appeal and delays spoilage, thereby improving shelf life (Armghan Khalid et al., 2022).

**Soy protein**, derived from soy flour or milk, offers a strong nutritional profile that rivals whey protein. Its exceptional adhesiveness, cohesiveness, and viscoelasticity enable the creation of transparent, flexible films. However, its hydrophilic nature limits its effectiveness as a water vapor barrier, posing challenges for broader food packaging applications. Additionally, soy protein shares allergenic properties with gluten, which can restrict its commercialization potential (Ahmad et al., 2021; Chen et al., 2019). Despite these challenges, edible coatings made from soy protein isolate—when combined with sodium ascorbate (an antioxidant) and glycerol (a plasticizer)—have proven effective in preserving the sensory and physiological qualities of fresh-cut apples for up to seven days at 10 °C (Hadidi et al., 2022).

In addition to soy protein, egg white protein, and zein—a prolamin protein extracted from maize—show significant promise in food preservation. Egg white protein extends shelf life by delaying ripening, preventing dehydration, and reducing microbial contamination (Jung et al., 2020). Zein, characterized by its hydrophobicity due to a high concentration of non-polar amino acids, forms highly effective films. The incorporation of fatty acids can further enhance its water vapor barrier properties, thereby increasing its suitability for edible film applications (Hassan et al., 2021). Each protein presents unique properties and challenges, creating exciting opportunities for developing advanced edible coatings with improved functionality and performance.

**Gelatin**, a protein obtained through the hydrolysis of collagen, plays a pivotal role in the food industry due to its exceptional film-forming and barrier properties. Renowned for its high transparency and mechanical strength, gelatin-based coatings are particularly effective in preserving perishable products by reducing water vapor transmission and delaying oxidation, thereby enhancing quality and shelf life. For instance, Gelatin-based edible coatings with *Mentha pulegium* essential oil (MEO) effectively preserved strawberries' physicochemical, microbiological, and sensory qualities during 13 days of refrigeration. The coating significantly reduced microbial growth, slowed physicochemical changes, and maintained firmness and color, with the 1 % MEO concentration showing the most protective effect. This bioactive coating offers a pesticide-free alternative for extending strawberry shelf life (Aitboulahsen et al., 2018). Similarly, Alparslan et al. (2016) investigated the antimicrobial activity of gelatin-based films with orange leaf essential oil against foodborne bacteria using the agar well-diffusion method. Films with 2 % essential oil showed the highest inhibition, with zones of 14.5 mm for *S. aureus* and 19.0 mm for *E. coli*. Yanwong and Threepopnatkul (2015) studied fish skin gelatin films with peppermint and citronella oils, achieving over 80 % growth inhibition of *E. coli* and *S. aureus* at 10 % oil concentration. The differences in antimicrobial effectiveness between Gram-negative and Gram-positive bacteria are attributed to the thinner peptidoglycan layer in Gram-negative bacteria. Essential oils demonstrated significant antibacterial potential in gelatin-based films. These applications underscore gelatin's versatility and efficacy as a sustainable solution for food preservation.

**Casein**, a protein derived from milk, exhibits remarkable film-forming capabilities and provides excellent oxygen barrier properties, making it an ideal choice for edible coatings. To enhance its flexibility and antimicrobial properties, casein is often combined with plasticizers and bioactive agents. These coatings are particularly effective for extending the shelf life and preserving the quality of dairy products, fruits, and baked goods. For instance, Murmu and Mishra (2018) developed caseinate and Arabic gum coatings with cinnamon and lemongrass essential oils (1 %–2 %) for guava, creating a semi-permeable film. The formation of covalent and hydrogen bonds between the casein, gum, and essential oils led to improved barrier properties by reducing free volume, hindering gas and water vapor transport. Ranjbaryan et al. (2019) found that the hydrophobic nature of the oils reduced water vapor permeability in caseinate films. The type of essential oil influenced the film's structure, with cinnamon oil forming a homogeneous structure, while ginger oil caused roughness. The incorporation of oils improved the uniformity and smoothness of the films but reduced their tensile strength. Using essential oils in nanoliposome form improved film structure and barrier properties by preventing excessive oil evaporation during drying. In a study by Apriliyani et al. (2021), the effectiveness of casein-chitosan edible coatings in maintaining the quality of broiler meat during refrigeration at 8 °C was evaluated over varying storage times (0 to 168 h). The study examined parameters such as water activity (Aw), cooking loss, organoleptic properties, physicochemical characteristics, and microbial growth (TPC, *S. aureus*, *E. coli*, and Salmonella sp.). The results demonstrated that casein-chitosan coatings significantly improved moisture retention, pH, lightness, and reduced microbial contamination, highlighting their potential to enhance food safety and extend the shelf-life of broiler meat. These examples underscore the versatility and effectiveness of casein-based coatings in enhancing food preservation across diverse applications.

### 2.3 Lipid-based coatings

Lipid-based coatings provide superior protection against environmental stressors, surpassing the effectiveness of polysaccharides and proteins due to their hydrophobic molecular structure. The natural water-repellent properties of lipids form a strong barrier that significantly reduces moisture permeation, helping to minimize water loss in perishable food items and ultimately prolonging their shelf life. These coatings exhibit remarkable compatibility when combined with other biopolymers, enhancing their functionality and enabling the delivery of bioactive ingredients (Devi, Jaiswal, & Jaiswal, 2024; Vakili-Ghartavol et al., 2024; Wu, Wu, & Hu, 2024). However, despite these advantages, lipid coatings are prone to oxidative rancidity due to exposure to atmospheric oxygen, resulting in undesirable sensory characteristics. Furthermore, lipids lack inherent film-forming capabilities and adhesive properties, limiting their direct application on certain food surfaces. These shortcomings can be overcome by combining lipids with other biopolymers and fillers, which improve their film formation, adhesion, and flexibility. The hydrophobic nature of lipid coatings is also difficult, as they are hard to dissolve with water, and their translucent, stiffer covering prevent attachment to fresh fruits and vegetables (Devi, Jaiswal, & Jaiswal, 2024). Lipid-based coatings, encompassing resins, vegetable oils, fatty acids, and waxes, have garnered significant attention in food preservation. Among these, **waxes** stand out due to their composition of straight-chain derivatives of alcohols and aliphatic acids, which impart exceptional efficacy in serving as water vapor barriers, thus enhancing food products' shelf life and stability.

**Natural waxes** such as carnauba, beeswax, paraffin, and candelilla wax are commonly used in food coatings to prevent water migration and reduce weight loss in fruits and vegetables (Vakili-Ghartavol et al., 2024). Beeswax is commonly used as a protective coating for citrus fruits like lime, mandarin, oranges, and apples (Sousa et al., 2021). This natural wax not only enhances the visual appeal of the fruits but also acts as a preservative, maintaining key physiological attributes such as vibrant pigments, aromatic compounds, and freshness. Carnauba wax also acts as an excellent carrier for bioactive ingredients, ensuring gradual and controlled delivery while maintaining the stability of the coating (Oliveira Filho et al., 2022). These lipid-based coatings not only improve the shelf life and quality of food but also enhance consumer acceptance by maintaining fresh produce's visual and sensory attributes.

In addition to waxes, **Vegetable oils** emerge as a significant category of lipid-based coatings that promise to enhance food preservation. Comprising mainly triglycerides, these oils contain small amounts of hydrocarbons, polyphenols, pigments, antioxidants, and phospholipids (Pandey et al., 2022). Derived from widely cultivated and cost-effective crops such as sunflower, mustard, olive, and corn, vegetable oils present a compelling alternative to traditional coating materials. Their eco-friendly nature is complemented by therapeutic properties that offer various health benefits. Research has demonstrated the practical effectiveness of oil-based coatings, particularly when combined with filler materials for fresh-cut fruits and vegetables, highlighting their substantial commercial potential (Gago et al., 2019). **Vegetable oils** are rich in various fatty acids, including unsaturated, saturated, short-chain, and long-chain. Research indicates that long-chain polyunsaturated fatty acids are promising for food coating applications (Nguyen et al., 2021). When used as surface coatings, these oils create a robust barrier against gas exchange, which helps maintain the freshness and quality of food products. However, this protective layer can also limit the release of distinctive aroma compounds. To address this issue, incorporating fatty acids into lipid-based coatings can serve as volatile precursors, promoting the formation of aromatic compounds that enhance the food's flavor profile. The concentration of fatty acids within these coatings is vital for controlling moisture permeability. Their non-polar nature helps reduce dehydration, protecting against chilling injury in refrigerated foods and freeze burn in frozen items. For example, meat coated with fatty acids before refrigeration or freezing demonstrates greater acceptability compared to uncoated versions (Arnon-Rips et al., 2019; Barzegar et al., 2024). This underscores the multifunctional benefits of lipid-based coatings in improving food quality and extending shelf life.

In lipid-based edible coatings, the reaction between acetic anhydride and acetylated glycerol yields 1-stearodiacetin, a compound that significantly enhances coating performance (Arnon-Rips et al., 2019; Arnon-Rips et al., 2021). The lipids produced from this reaction can stretch up to 102 % of their original length before breaking, showcasing remarkable elasticity. Notably, acetylated glycerol monostearate can extend up to an impressive 800 %. These coatings also exhibit much lower vapor permeability than other materials, making them highly effective in moisture control. This property is particularly beneficial for preserving poultry and meat products, where moisture retention is essential for maintaining quality (Nda-Umar et al., 2020).

**Lipid-based edible coatings** frequently incorporate **resins**—organic polymers known for their unique properties. Unlike traditional lipids, resins dissolve in alcohol and alkaline solutions and are released by plants when injured or infected. These semisolid, transparent substances are insoluble in water but dissolve in organic solvents, making them particularly useful in food and agricultural applications (Tanwar et al., 2024). When included in edible films, resins provide a glossy finish to fruits and vegetables, enhancing their visual appeal. Beyond aesthetic enhancements, resins significantly lower the permeability of gases such as ethylene, oxygen, and carbon dioxide—crucial factors for preserving the freshness of agricultural products. Shellac is a commonly used resin for coating citrus fruits; however, it is not classified as Generally Recognized as Safe (GRAS) and is approved only as an indirect food additive. Despite this limitation, shellac coatings effectively reduce surface pitting and minimize postharvest losses, illustrating their practical value in the food industry (Reichert et al., 2020).

### 2.4 Composite and hybrid coatings

Developing composite or multi-layered edible coatings is a transformative approach to food preservation, overcoming the limitations of individual compounds and harnessing their synergistic benefits. These coatings, which typically combine lipids and hydrocolloids, are specifically designed to enhance moisture retention, improve mechanical strength, and extend the shelf life of perishable food products. By using combinations such as carbohydrates and proteins, proteins and lipids, or carbohydrates and lipids, these composite coatings deliver superior mechanical properties, with the coating's performance primarily determined by the specific carbohydrate or protein used (Fang et al., 2025; Gautam et al., 2024).

**Shellac-tannic acid** coatings have been shown to significantly prolong the shelf life of mangoes at room temperature, preserving both their firmness and reducing weight loss. Incorporating tannic acid in the coating enhances its antifungal activity, extending the fruit's storage life. Similarly, a whey protein-xanthan gum coating, combined with clove oil, effectively preserved tomatoes by improving the retention of sugars and phenolics, inhibiting microbial growth, and reducing the respiration rate, extending their shelf life to 15 days at 20 °C (Sharma, Goyal, et al., 2024; Taghizadeh et al., 2024).

**Multi-layered antimicrobial** coatings have proven effective in preserving the texture and quality of fruits like pineapples by reducing microbial contamination during storage. Likewise, composite coatings made from rice starch, carrageenan, and sucrose fatty acid esters have preserved the phytochemical properties of plums during storage. Further, chitosan coatings grafted with chlorogenic acid have demonstrated the ability to inhibit decay and maintain ascorbic acid content in peaches for up to 8 days at 20 °C (Jiang et al., 2024; Rossi-Márquez et al., 2023).

The potential of composite edible coatings is further exemplified in applications like preserving fresh-cut mangoes with coatings comprising sesame protein, guar gum, calcium chloride, and mango puree, which extended shelf life and preserved color and sugar content. Similarly, coatings on red guavas formed modified atmospheric conditions around the fruit, slowing ripening and maintaining firmness and green color (Silva et al., 2021). Additionally, bi-layer coatings of citrus fruits, such as mandarins and navel oranges, significantly improved glossiness and flavor retention. Beyond fruits and vegetables, composite coatings have also shown promise in meat preservation. For example, an alginate-based edible coating with nisin-palmitoyl successfully inhibited the growth of *Staphylococcus aureus* in beef slices (Ramdan et al., 2023). In dairy, composite coatings combining chitosan with other polysaccharides like starch, cellulose, and gelatin offer protection from UV rays and temperature fluctuations, enhancing sensory properties in cheeses, especially when probiotics are included in the coating. This innovation underscores the critical role of composite edible coatings in meeting modern food preservation and quality demands.

## 3 Innovations in coatings and their physicochemical and functional properties

Advanced technologies in edible coatings are revolutionizing food preservation, extending beyond traditional methods like casting, extrusion, dipping, and spraying (Fig. 8). These foundational techniques are now enhanced by cutting-edge approaches such as multilayering and nano structuring, which significantly improve the protective capabilities of edible coatings. Multilayering adds complexity and increases the durability of the coating, while nano structuring offers unparalleled control at the molecular level, providing superior protection against environmental factors (Thamarsha et al., 2024). Furthermore, developing coatings with advanced properties, such as active, self-healing, and superhydrophobic characteristics, transforms food packaging. Active edible coatings, integrated with antimicrobial or antioxidant agents, offer enhanced food safety by combating microbial growth and oxidation. Self-healing coatings add a layer of resilience, automatically repairing damage to maintain the protective barrier. With their ability to minimize water transfer, superhydrophobic coatings dramatically improve moisture resistance, ensuring longer shelf life and freshness (In Kim et al., 2024). This Fig. 6 highlights key active components and their natural sources for enhancing edible coatings' effectiveness in food quality and safety.

Biopolymers, including widely studied hydrocolloids like alginates and chitosan, are the primary materials for these innovative coatings. These biopolymers, whether protein-based or polysaccharide-based, are inherently hydrophilic, making them ideal candidates for food packaging. However, their properties can be further enhanced through reinforcement with nanoparticles, essential oils, and bio-nanocomposites. These additions improve the physicochemical characteristics of the coatings, offering excellent protection, functionality, and sustainability (Li, Cheng, et al., 2024).

### 3.1 Essential oils

Essential oils (EOs), naturally derived aromatic compounds extracted from various plant parts such as seeds, stems, leaves, flowers, and fruits, present a compelling solution for enhancing food preservation. These EOs are widely recognized as Generally Recognized as Safe (GRAS) antimicrobial additives, making them ideal candidates for incorporation into edible coatings. Their potent antimicrobial properties effectively combat foodborne pathogens, protecting materials like fruits, vegetables, and meat products from spoilage. Due to their hydrophobic nature, EOs significantly enhance the hydrophobicity of edible coatings, thereby reducing moisture vapor penetration and improving shelf life (Devi, Das, et al., 2024; Felicia et al., 2024). Apart from their antibacterial and preservation properties, essential oils assist in maintaining the physical stability of active substances, hence conserving their flavor, fragrance, and taste, particularly when added to nano-emulsions. These nano-systems stabilize EOs and optimize their effectiveness against foodborne pathogens, offering a highly efficient delivery system that minimizes sensory disruption in food products. Using nano-emulsions as delivery vehicles ensures that the volatile compounds in EOs maintain their efficacy while avoiding interference with the sensory attributes of food (Silva Cesca et al., 2024).

Moreover, integrating EOs into suitable delivery systems enhances their stability and protective effect, reducing reliance on traditional preservatives. EOs such as cinnamon, oregano, and thyme have been extensively studied for their antimicrobial and antioxidant properties across various food matrices, demonstrating their potential to revolutionize food preservation technologies. Incorporating EOs into edible coatings offers a sophisticated, sustainable approach to extending the shelf life of perishable food products while safeguarding both quality and safety (Alonso et al., 2024).

Despite the potential benefits, the use of essential oils in food packaging applications presents several drawbacks (Alparslan et al., 2016). One significant challenge is their volatility and strong aroma, which can alter the sensory properties of food, affecting its taste and smell. Additionally, essential oils can be expensive, which may limit their scalability for mass production (Murmu & Mishra, 2018). Their antimicrobial properties, while beneficial, can sometimes be too intense, leading to undesirable effects on the texture and quality of certain food products. Furthermore, the instability of essential oils, particularly under varying environmental conditions like temperature and light, can reduce their effectiveness over time. Finally, the potential for allergic reactions or sensitivities to specific essential oils in consumers may limit their widespread acceptance in food packaging (Yadav, Kumar, Upadhyay, Sethi, & Singh, 2022; Yadav, Kumar, Upadhyay, Singh, et al., 2022; Yu et al., 2023).

### 3.2 Nano-emulsions

The use of nano-emulsions, water-in-oil, or oil-in-water solutions is an innovative and novel approach to improve the physicochemical qualities of edible coating formulations. Oil-in-water nano-emulsions, in particular, are ideal for this application due to their compatibility with food-grade components, facilitating more effortless scalability during production. The mechanism behind nano-emulsions involves the formation of nanodroplets (ranging from 10 to 100 nm) coated with a film or layer of food-grade ingredients, enhancing both bioavailability and chemical reactivity. The reduction in droplet size increases the surface area, amplifying the functional properties of encapsulated components, such as antibacterial essential oils (EOs), and improving the absorption of hydrophobic compounds. This enhanced surface area correlates with superior antibacterial efficacy and better shelf-life extension. Furthermore, the optical clarity of these droplets ensures that the coatings remain visually appealing while retaining their effectiveness (Iqbal et al., 2024).

Integrating EOs into edible coatings via nano-emulsions is highly efficient since it allows for the use of small amounts of EO while not severely compromising the sensory properties of the food. The nanoscale characteristics of these emulsions ensure the requisite shelf life extension. Oil-in-water nano-emulsions have the potential to revolutionize edible coatings by providing a homogenization-enabled, scalable solution for the food sector. There are two common methods for incorporating nano-emulsions into edible coatings: the single-step process, in which all ingredients are mixed and homogenized to create nanometric droplets, and the two-step process, in which the aqueous solution is prepared first and then combined with the biopolymer solution. Nanoemulsions have demonstrated remarkable potential in preserving a wide range of postharvest fruits, including papaya, mango, and strawberries, demonstrating their practical applicability in the food business (Ashrafi et al., 2024; Filho et al., 2021).

### 3.3 Bio-nanocomposites

Bio-nanocomposites are an advanced amalgamation of diverse nanocomponents systematically arranged to build robust barriers against the penetration of molecules such as oxygen and water vapor, hence minimizing weight loss and improving material durability. The efficacy of polymer-based nanocomposites is significantly influenced by the concentration of the composite ingredients, with ideal ratios yielding enhanced performance. Furthermore, research has demonstrated that a homogeneous dispersion of nanofillers within the composite structure results in enhanced bio-nanocomposite properties, improving overall functionality (Paidari et al., 2023).

When applied to developing edible coatings, nanocomposites bring critical enhancements, particularly in biopolymers' mechanical strength, barrier capabilities, thermal stability, and antimicrobial properties. For instance, the incorporation of silver nanoparticles (Ag NPs), chitosan nanofibers, titanium dioxide (TiO2 NPs), Zein NPs, cellulose nanofillers, and copper oxide nanoparticles (CuO NPs) significantly boosts water barrier properties, reducing solubility and increasing contact angle with water (Hassanen et al., 2023). This is accomplished by constructing a convoluted pathway that constricts pore channels, elongating the diffusion pathway and impeding water transport. The homogeneous distribution of these nanofillers produces an enhanced gas barrier effect by forming intricate channels that obstruct the unrestricted flow of gas molecules. The exact arrangement of nanofillers modifies the interfacial characteristics of the polymer matrix, hence improving its resistance to gas permeation. These advancements make bio-nanocomposites indispensable in creating high-performance edible coatings with improved shelf life and functionality (Li et al., 2023).

### 3.4 Inorganic nanoparticles

Solid colloidal particles with a size between 10 and 100 nm are known as inorganic nanoparticles (NPs), and they are unique in their stability, effectiveness, and physiological function. These nanoparticles have the unique ability to encapsulate functional molecules, thereby enhancing their stability and performance. The primary methods for synthesizing NPs include polyelectrolyte complexation, precipitation, covalent crosslinking, and ionic crosslinking, each offering distinct advantages for tailoring particle characteristics. NPs exhibit potent antimicrobial properties, making them an ideal candidate for protective applications in food systems. Their incorporation into food matrices enhances preservation by preventing microbial growth while minimizing flavor alterations (Ozuna-Valencia et al., 2024).

Furthermore, the high surface area of NPs contributes to improved reinforcement within the matrix, resulting in more effective and uniform distribution. The versatility of NPs in food applications lies in their capacity for easy dispersion into edible matrices, ensuring that their antimicrobial properties are delivered without compromising food sensory qualities (Roy et al., 2024). This leads to enhanced diffusion and bioavailability of active compounds while mitigating the negative impact on food odors typically associated with conventional antimicrobial agents. Consequently, integrating NPs into edible coatings is a highly effective strategy to improve food safety and shelf life without sacrificing the consumer's sensory experience.

### 3.5 Bio-printing or 3D food printing

3D food printing, or bio-printing, represents a ground breaking technology with significant potential to revolutionize the food industry. It involves creating intricate food structures by depositing ingredients layer by layer, allowing for precise control over food composition, texture, and nutritional content. One of its most promising applications is in the creation of edible food coatings, which are vital for preserving the quality, freshness, and safety of food products (Andriani & Handayani, 2023). By enabling the customization of coatings to meet specific needs, 3D food printing can provide solutions that traditional coating methods cannot.

The process of 3D food printing involves three core components: food materials, a 3D printer, and a control system. Ingredients such as pureed fruits, vegetables, proteins, and plant-based substances are extruded through a nozzle, where they are deposited in a precise pattern to form a three-dimensional structure (Hakim et al., 2024). The rheological properties of the ingredients—such as flow and viscosity—are crucial for effective printing. The use of edible inks made from natural components like proteins, carbohydrates, and lipids ensures that the printed food is both safe and consumable. When applied to edible coatings, 3D food printing offers the ability to design coatings with custom shapes and textures, enabling new functionalities like controlled release of preservatives or the creation of protective barriers that extend shelf life and enhance food quality (Andriani & Handayani, 2023; Hakim et al., 2023).

Edible coatings play an important role in reducing food spoilage by serving as a barrier to external factors such as moisture, oxygen, and microbial contamination. The precision of 3D printing allows for the creation of coatings that are perfectly suited to the shape and surface area of various food items, ensuring even coverage and optimal protection (Wang, Gao, et al., 2024; Wang, McClements, et al., 2024). For instance, chitosan-based edible coatings, which have inherent antimicrobial properties, have been explored in combination with 3D printing for fruits and vegetables. These coatings can reduce moisture loss and inhibit bacterial growth, significantly extending the shelf life of perishable items (Deng et al., 2020; Xing et al., 2016). In addition to enhancing preservation, 3D food printing offers the possibility of incorporating functional ingredients into food coatings. This could include vitamins, antioxidants, or other nutrients, enabling the creation of coatings that not only preserve food but also enhance its nutritional profile. A study by Xie et al. (2023) demonstrated the use of 3D printing to embed micronutrients into edible coatings for fresh produce, combining both preservation and health benefits in one application.

One of the most compelling aspects of 3D food printing is the ability to tailor food products to individual consumer preferences. In the context of edible coatings, this can mean customizing the flavor, texture, or appearance of food to match consumer desires. For example, researchers (Waseem et al., 2024) have developed 3D-printed coatings for baked goods that create a crispy texture while improving the visual appeal of the food. This level of customization opens up possibilities for new eating experiences and personalized food products, which is a growing trend in the food industry. Furthermore, 3D food printing has the potential to address global concerns about food waste. By utilizing ingredients that might otherwise be discarded, such as surplus fruits and vegetables, 3D printing can create coatings from renewable resources like algae (Deng et al., 2020; Hakim et al., 2023). These sustainable coatings not only reduce food waste but also promote environmentally friendly food production practices.

Despite its potential, the widespread adoption of 3D food printing for edible coatings faces several challenges. The high cost of 3D printers, ingredients, and the need for specialized knowledge in food material science pose barriers to broader implementation. Additionally, balancing functional properties with sensory qualities such as taste and texture remains a challenge that must be addressed to ensure consumer acceptance. Looking ahead, the future of 3D food printing in edible coatings is promising (Andriani & Handayani, 2023). As advancements in material science, printing technology, and ingredient formulations continue, 3D food printing is poised to become a mainstream tool in the food industry. It holds the potential to transform how edible coatings are designed and produced, offering more efficient, personalized, and sustainable solutions for food preservation. As research and development in this field progress, 3D food printing is likely to play a key role in shaping the future of food production and packaging (Wang, Gao, et al., 2024; Wang, McClements, et al., 2024).

## 4 Exploring functional benefits of edible coatings and films

Biopolymer-based edible films and coatings are transforming the sustainable packaging industry by eliminating the boundaries between packaging, preservation, and food. These innovative films, which are biodegradable, moisture-resistant, and entirely edible, offer a promising solution for protecting perishable foods. By encapsulating food in these coatings, we can prevent color fading, lipid oxidation, and off-odors, significantly enhancing the shelf life and quality of products, especially animal-based items (Bizymis et al., 2024; Thamarsha et al., 2024).

Even more exciting is the ability to incorporate active compounds such as nutraceuticals, enzymes, vitamins, flavorings, phenolic compounds, and organic acids into these films, elevating their functionality and extending their usefulness in real-world food systems. These advancements have expanded the potential applications of edible coatings, offering food manufacturers a sustainable, health-conscious alternative to traditional packaging methods (Thamarsha et al., 2024). The Fig. 7 illustrates the key steps in edible film formation, including material preparation, processing, and final film drying stages.

### 4.1 Shelf-life extension

Increasing the shelf life of food is a top priority in the food processing and preservation industry. As consumer lifestyles rapidly change, people face time constraints when preparing food due to work-life imbalances. This has led to a significant rise in demand for shelf-stable foods. Active packaging solutions have become essential in prolonging shelf life and enhancing the organoleptic properties of food (Zhao et al., 2024). Nutrient-rich foods, which are ideal for microbial growth, can spoil when microbes proliferate, producing toxins and deteriorating food quality. To address this, plant-based edible coatings are gaining attention as an effective solution to prevent food spoilage.

These naturally sourced coatings act as protective barriers on food surfaces, preventing gas exchange and moisture loss. They provide an eco-friendly alternative to traditional packaging methods. When infused with antimicrobial agents and antioxidants, these coatings offer dual benefits: they slow spoilage caused by microbes and prevent changes like enzymatic browning (Kulawik et al., 2024). This helps maintain the quality and freshness of food while preserving its nutritional content. Additionally, the biodegradable nature of these films meets the rising consumer demand for environmentally friendly packaging options. The slow-release properties of active substances in the coatings improve preservation by limiting pathogen growth on the food surface. The active ingredients in these coatings—such as flavoring compounds, antioxidants, antimicrobial agents, pigments, and nutrients—enhance the organoleptic and nutritional properties of food. Biopolymer-based edible films, including vitamins, plant extracts, phenolic compounds, and essential oils, are efficient carriers for these ingredients, aiding in food preservation (Yadav, Kumar, Upadhyay, Sethi, & Singh, 2022; Yadav, Kumar, Upadhyay, Singh, et al., 2022). Gums, like those used in fruit and vegetable coatings, offer affordability, biocompatibility, and effective control over maturation. Ongoing research continues to refine plant-based coatings, improving their barrier, antimicrobial, and antioxidant properties to meet the growing demand for extended shelf life and sustainable packaging (Abdi et al., 2023).

Recent studies highlight the effectiveness of plant-based edible coatings in extending shelf life. For example, garlic extract in a zein-based coating successfully inhibited mold growth on bread for 30 days (Almeida et al., 2024). Similarly, coatings with ginger and garlic extracts, combined with *aloe vera* and gum Arabic, reduced weight loss and spoilage in guava fruits during storage (Li, Zhou, et al., 2024). In other studies, starch-coated strawberries had a 66 % longer shelf life compared to uncoated ones, and lime fruits coated with a biopolymer film showed reduced wilting and color loss at higher temperatures (Costa et al., 2023). These findings emphasize the potential of plant-based edible coatings as an innovative and sustainable solution for preserving food quality and extending shelf life.

### 4.2 Antioxidant properties

Plant-derived coatings combined with antioxidant chemicals offer an efficient and eco-friendly alternative for food packaging. The antioxidants are integrated into the film or coating, allowing for a controlled release that helps prevent food oxidation, thus extending shelf life. By including antioxidants, the coatings improve their functional properties and reduce the negative effects of oxidation, such as changes in mouthfeel, color, aroma, flavor, and texture. Antioxidants neutralize free radicals and reactive oxygen species (ROS), which can otherwise damage food components like lipids, proteins, and pigments, affecting food quality and safety (Aguado et al., 2024; Giacondino et al., 2024; Raheena & Shankar, 2024; Tran et al., 2024).

Plant-derived antioxidants play a key role in the emerging field of edible coatings, using bioactive compounds such as anthocyanins, tannins, polyphenols, vitamins C and E, and carotenoids (Lourenço et al., 2019). These compounds are extracted from natural sources like tea leaves, leafy greens, citrus fruits, and soybeans. They have great potential to improve food preservation and quality. Among them, ascorbic acid (vitamin C), commonly found in citrus fruits and berries, stands out for its dual function. It not only neutralizes harmful free radicals but also regenerates other antioxidants, like α-tocopherol (vitamin E) (Xylia et al., 2021). This interaction enhances the protective effects, with α-tocopherol being especially valuable in food packaging for preventing lipid oxidation and preserving the quality of fats and oils. Polyphenols, including flavonoids like quercetin, phenolic acids such as gallic acid, and stilbenes like resveratrol (Yu et al., 2023), are also important. These compounds, known for their strong antioxidative properties, work together with carotenoids like β-carotene and lycopene to protect against oxidative damage and enhance the visual appeal of food products.

Tannins, found in seeds, nuts, and some fruits, offer added protection through their antioxidative and antimicrobial properties. These natural antioxidants improve the effectiveness of edible coatings used for food preservation. They help extend shelf life, enhance safety, and improve sensory qualities by reducing oxidative and microbial spoilage. As a barrier against moisture and oxygen, these coatings combine scientific innovation and practical food technology, providing a sustainable solution to modern food preservation (Monasterio et al., 2023). To maximize their benefits, the concentration, stability, and extraction methods of these antioxidants need to be carefully optimized. While synthetic antioxidants such as BHT, BHA, PG, and TBHQ are commonly used for their stability, performance, and cost-effectiveness, plant-based alternatives are becoming more popular due to their natural origin and consumer demand for sustainability.

Oyom et al. (2022) introduced an innovative food preservation method by creating an edible film and coating made from modified sweet potato starch and fortified with 0.2–0.4 % cumin essential oil. This new formulation improves the color, barrier properties, and mechanical strength of starch-based films. It has shown promise in preserving fruit pulp, reducing degradation, and inhibiting enzymatic activity during 28 days of storage under ambient conditions. The coating also boosts the activity of key antioxidant enzymes, which help preserve fruit firmness and slow down softening. The coated samples outperformed untreated controls in resisting spoilage, indicating the coating's potential to redefine food preservation standards. Ezati et al. (2022) demonstrated that using cellulose nanofiber combined with nitrogen-functionalized carbon dots (N-CDs) from glucose on tangerines and strawberries extended their shelf life by over 10 days and 2 days, respectively, by significantly inhibiting fungal growth. The integration of carbon dots also enhanced the coatings' UV-blocking ability while maintaining optical transparency. These coatings showed improved water vapor permeability, increased contact angles, and notable antioxidant activity. Further studies revealed that carboxymethyl cellulose-based coatings enriched with bioactive compounds from spent coffee grounds (SCG) extended the shelf life of fresh goldenberries. The coatings also boosted the fruit's antioxidant activity, thanks to the phenolic compounds in SCG and the polysaccharide-rich extract in the edible coating, demonstrating their potential for preserving goldenberries under various storage conditions (Galindez et al., 2021). These findings highlight the growing potential of innovative edible coatings in extending the shelf life of perishable fruits and enhancing their nutritional value.

### 4.3 Biodegradable

Conventional plastic packaging poses a major environmental challenge, with the food sector being a key contributor to global plastic waste. In response to growing concerns about the impact of synthetic polymers, the use of biodegradable alternatives is increasing. This has led to extensive research into new sources of biodegradable packaging materials (Oraç et al., 2023). One promising innovation is the development of edible films with improved properties such as water resistance, transparency, lightness, strength, and flexibility. Biopolymers derived from natural sources like polysaccharides, lipids, and proteins are becoming viable alternatives to traditional plastic packaging. Seed gums, in particular, are gaining attention for their excellent material properties and biodegradability. Various seed gums, including those from Mesquite, Flax, Chia, Psyllium, Cassia, Tamarind, Qodumeshirazi (Alyssum homolocarpum), Wild Sage, Cress, *Lepidium perfoliatum*, Durian, Basil, Balangu, and *Dracocephalum moldavica*, have shown favorable mechanical, physical, thermal, and microstructural properties that make them suitable for food packaging (Eftekhari et al., 2023). These natural materials provide an eco-friendly solution to plastic waste while offering the necessary durability and functionality for food preservation. The potential of seed gums to replace synthetic packaging materials marks an important step towards more sustainable, biodegradable packaging solutions in the food industry.

### 4.4 Antimicrobial properties

Food processing and packaging have prioritized food preservation, focusing on minimizing microbial contamination, particularly as the food industry experiences rapid growth. Fruits and vegetables, from the moment they are grown to when they are sold to consumers, are vulnerable to contamination. This can occur at various supply chain stages—during cultivation, harvesting, handling, transport, storage, and even at the point of sale. Furthermore, contamination can persist even after the consumer has made a purchase. Several factors, including the type of produce, cultivation practices, geographic location, and climate conditions before harvest influence the microbial load on fresh produce (Hafezi et al., 2024; Rout & Pradhan, 2024).

Microbial spoilage is not only a threat to food safety but also alters its texture, color, aroma, flavor, and nutritional value. The primary culprits of spoilage include bacteria, fungi, yeasts, and molds, though factors like moisture, temperature, pH, osmotic pressure, salinity, and the gaseous environment can further promote microbial growth. A promising solution to this problem is incorporating antimicrobial compounds into food packaging, a key component of active packaging technology (Yemenicioğlu, 2024). These antimicrobial agents, seamlessly integrated into the polymer matrix, form films and coatings that directly interact with the food surface, creating a barrier against spoilage-causing microbes. The Fig. 8 illustrates the mechanisms by which antimicrobial coatings in food packaging inhibit pathogen growth, highlighting their potential to enhance food safety and extend shelf life through active surface protection.

However, as consumers become increasingly aware of the harmful effects of synthetic antimicrobials, there is a growing demand for natural, plant-based alternatives. Synthetic compounds like sulfite-based additives and benzoic acid derivatives are being rejected due to health concerns, including the degradation of essential nutrients such as vitamin B1 (thiamine). This shift towards plant-based, sustainable materials not only addresses consumer health concerns but also offers a promising avenue for the future of food preservation (Betancur-D'Ambrosio et al., 2024; Vakili-Ghartavol et al., 2024).

Introducing antimicrobial chemicals straight into coatings has advantages over dipping, brushing, and spraying (Fig. 9). This approach results in a more uniform dispersion and allows for the regulated, prolonged release of antibacterial chemicals, which improves food product protection. However, the constant use of antimicrobial agents in coatings contribute to developing microbial resistance, necessitating continuous innovation and testing of new antimicrobial formulations. This challenge underscores the importance of ongoing research to develop effective, resistance-free antimicrobials that can adapt to evolving microbial threats (Ru et al., 2024).

One significant problem in employing natural antimicrobials is their intrinsic instability, low dispersing within foods, and the potential to contribute unpleasant odors. Consequently, extensive research focuses on identifying and utilizing plant-based antimicrobials, which are considered safer alternatives to synthetic chemicals. Plant-derived antimicrobials fit within the framework of food safety measures and target foodborne pathogens through mechanisms that inhibit microbial growth and disrupt cellular structures. Common antimicrobial plant compounds include polyphenols and essential oils, which have proven effective against foodborne bacteria (Chau et al., 2024).

Plant-derived antimicrobial compounds likely hold considerable potential, drawing from many phytochemicals. These include terpenoids such as carnosic acid, polyphenols like quercetin, and sulfur-containing compounds like thiols (e.g., allicin) (Zhu, 2021). Essential oils are abundant in different plant parts—such as seeds, leaves, stems, buds, and flowers—include compounds like carvacrol, citral, and linalool, showcasing their diverse antimicrobial properties. Rosemary essential oil, for example, contains monoterpenes such as α & β pinene, borneol, and camphor, all of which have potent antimicrobial properties (Nawaz et al., 2020). Polyphenols extracted from industrial wastes like pomegranate peels, green tea, and coffee pulp have also demonstrated effectiveness against common spoilage organisms like *Salmonella typhimurium*, *S. aureus*, and *Escherichia coli*. Incorporating such plant phenolics into palatable coatings provides increased stability against oxidation, extending the shelf life of food goods (Khojah, 2020).

Wang et al., (Wang et al., 2019) developed an antimicrobial liquid-based preservative in 2019 by incorporating nano-meter-sized silver and ginkgo leaf lipid components into a lacquer wax composite. This innovative formulation demonstrated a robust antibacterial-effect against *Bacillus subtilis*, *Staphylococcus aureus*, and various mold species (Wang et al., 2019). However, plant-based antimicrobials present a compelling alternative, offering advantages such as sustainability, biodegradability, low cost, and easy availability. In addition to adding economic value, sourcing these chemicals from not edible parts of food crops increases plant cultivation's profitability. Furthermore, plant-based edible coatings can address concerns about the diffusibility of antimicrobial compounds and facilitate controlled release, providing an efficient and sustainable method for food preservation (Fang et al., 2025).

Nevertheless, plant-based edible coatings face challenges primarily related to their susceptibility to environmental conditions like humidity and temperature. The stability of these coatings is critical; improper storage can lead to a loss of antimicrobial efficacy, as the compounds degrade over time. High temperatures can accelerate the diffusion rate of active molecules, while increased humidity can compromise the barrier properties, leading to a higher rate of microbial spoilage. As a result, ensuring the proper storage conditions is essential for maintaining the effectiveness of these natural preservatives, highlighting the need for ongoing research into enhancing the stability and performance of plant-based edible coatings under varying environmental conditions (Bouarab Chibane et al., 2019; Rivero-Pino et al., 2023).

### 4.5 Target delivery of nutrients

Essential nutrition is fundamental to maintaining health, but the bioavailability of essential nutrients in food can be limited by factors such as poor solubility, chemical instability, and undesirable taste. Nutraceuticals, including omega-3 fatty acids, carotenoids, and curcumin, are subject to potential degradation by digestive enzymes and the intestinal wall, which can compromise their bioavailability (Daniloski et al., 2021). These dietary supplements are substantiated by scientific evidence for their roles in promoting health, enhancing physiological functions, and mitigating disease risks, thus positioning them as valuable agents for nutritional and therapeutic purposes. Beyond their fundamental nutritional value, these bioactive compounds hold enormous potential for various applications, particularly in functional foods.

Over time, various bioactive compounds derived from food and plants, including plant polyphenols, vitamins, probiotics, polysaccharides, and peptides, have been developed into pharmaceutical-formulations such as solutions, gels, powders, and capsules. Epidemiological studies have shown the health-promoting effects of these compounds, especially those from plant sources (Thy et al., 2023). Despite their benefits, the direct consumption of nutraceuticals can be challenging, so integrating them into food products is essential for enhancing their accessibility and effectiveness. Advanced nutrient delivery systems—such as time-responsive, temperature-responsive, enzyme-responsive, and pH-responsive systems—are increasingly being used in both the food and pharmaceutical industries to optimize the delivery of these compounds.

Edible coatings and films offer an innovative solution to incorporate active nutraceutical ingredients into food products. Research has demonstrated the successful integration of nutraceuticals into these coatings, with ongoing studies aiming to determine optimal concentrations that do not compromise the films' fundamental properties, such as barrier and mechanical strength (Daniloski et al., 2021; Fernández-Luqueño et al., 2021; Hu et al., 2023). Micro- and nanoencapsulation techniques further enhance these compounds' stability and controlled release, making them ideal for application in a wide array of food products, thereby improving their nutritional value without altering their sensory characteristics.

### 4.6 Moisture barrier

Adequate packaging plays a crucial role in preserving food products' sensory qualities, overall quality, and shelf life, ensuring their commercial success. It is assumed that to minimize food waste and extend shelf life, packaging solutions should effectively control moisture levels. In the canning industry and alcoholic beverage packaging, glass and metal containers are the preferred choices, given their excellent ability to block gas and moisture (Dharhshini & Sistla, 2023). However, these materials present significant environmental challenges, including non-biodegradability in landfills, high energy costs for recycling, and elevated transportation expenses. Polysaccharide-based polymers can absorb or release moisture in reaction to varying environmental conditions, facilitating the maintenance of a dynamic equilibrium moisture content that adjusts to changes in temperature and humidity. This property enables a more responsive strategy for moisture level regulation (Azadbakht et al., 2021; Dharhshini & Sistla, 2023).

Fruits and vegetables continue to undergo biological processes postharvest, leading to water and solute loss, which impacts their quality. The moisture content of fresh produce can exceed 80 % at harvest, and even a small decrease in moisture can significantly heighten susceptibility to physiological disorders, such as shriveling and reduced shelf life. This makes the application of edible coatings immediately after harvest a common practice. Edible coatings provide an additional barrier layer, reducing the transfer of moisture and gases between the food and its environment, thereby preserving its quality (Qiu et al., 2019). Historically, fruit waxing in 12th-century China and meat larding in 16th-century England exemplified early attempts at using coatings for preservation, significantly reducing water loss and delaying senescence.

Modern edible coatings form a semi-permeable layer that controls moisture and gas exchange, preventing over-ripening, oxidative browning, and early maturation while avoiding anaerobic conditions that could lead to spoilage. Similar to modified atmosphere packaging (MAP), these coatings help retain the internal gas composition and regulate the respiratory rate of fresh produce (Tabassum & Khan, 2020). Since microbial growth and enzymatic reactions thrive in humid conditions, coatings with enhanced moisture barriers offer an innovative solution to extending shelf life. Thus, continued development of advanced edible coatings is critical for improving food preservation while minimizing environmental impact.

Tainting is a notable concern when storing fruits and vegetables with strong or pungent aromas. Edible coatings, particularly those with a bipolar matrix, are highly effective in mitigating this issue. Non-polar aromatic compounds, which are less likely to interact with hydrophilic proteins and polysaccharides, benefit from the hydrophobic nature of these coatings, making them efficient at combating odors (Chen et al., 2021). This property also extends to oils and grease, enhancing the overall preservation of the produce. As natural, sustainable, biodegradable, and compostable materials, edible coatings align with the growing demand for environmentally conscious solutions in the food industry. These plant-based coatings provide minimal health hazards and are classified as “Generally Recognized As Safe” (GRAS) for usage by humans. They are essential in extending food shelf life and reducing deterioration and waste products. Furthermore, they have demonstrated efficacy in improving the appearance and gloss of food goods, making them more visually appealing and marketable. While plant-based coatings provide a more sustainable alternative to synthetic alternatives, their performance attributes present unique issues (Attar et al., 2021). While these coatings generally provide good barrier properties, their effectiveness can be limited compared to synthetic alternatives, especially in high-moisture food systems. The polar compounds in plant-based materials can struggle with solubility, making them less effective for foods with high water content. For example, plant-based wax coatings lose flexibility at low temperatures and become brittle, compromising their ability to maintain coating integrity and increase moisture loss. Furthermore, coatings with lower melting points become softer at higher temperatures, decreasing their ability to act as a barrier.

A thorough analysis of nine different edible coatings made from pectin, cellulose nanocrystals, glycerol, and lemongrass essential oil was carried out by Da Silva et al. (da Silva et al., 2019) and applied to strawberries. According to the study, these coatings considerably reduced weight loss and maintained the fruit's pH levels, preserving its chemical and physical integrity over time. The effectiveness of plant-based coatings that contain asparagus waste extract on strawberries was also investigated by Liu et al. (Liu et al., 2021). Notable antifungal qualities against *Penicillium italicum* were found in their study, along with a delay in color deterioration and the retention of flavonoid and phenolic components. These results support the idea that these edible coatings are a safe, efficient, and environmentally friendly way to extend the shelf life of strawberries.

### 4.7 Endurance and aesthetic improvements

Wax coatings on fruits and vegetables enhance their glossiness and shine and provide an effective barrier against moisture and gases, ensuring improved shelf life and quality preservation. Waxing in the United States commenced in the 1930s with the application of paraffin wax on citrus fruits. In 1950, carnauba wax was incorporated for use on fresh fruits and vegetables (Miranda et al., 2021). The food industry is increasingly adopting plant-derived coatings in response to the growing demand for sustainable, biodegradable, and edible packaging. These coatings are engineered to replicate the desirable qualities of traditional waxes, such as providing organic barriers to gas and moisture (De Castro Silva et al., 2020). Research into coatings made from materials such as cellulose, casein, gluten, wheat, starch, and soy protein is gaining traction, as these biopolymers can deliver similar protective benefits while aligning with sustainability goals. These coatings preserve food quality and enhance its physical appearance by adding color, gloss, and a more appealing visual aesthetic (Choeybundit et al., 2024). For instance, mango kernel starch-based pouches have been shown to outperform traditional LDPE pouches in packaging red chili powder, with better retention of color and minimal reduction in capsaicinoid content (Yadav, Kumar, Upadhyay, Singh, et al., 2022). Such advancements highlight the potential of plant-based coatings to offer functional and aesthetic improvements, paving the way for a greener, more efficient approach to food packaging.

### 4.8 Structural properties

One of the key challenges in developing edible films is ensuring proper handling, as they often struggle with certain physical limitations. Plastic films have outstanding barrier qualities, strength, stiffness, and ease of handling, and they can maintain these properties at very thin microns. However, the growing demand for sustainable, biodegradable, and edible packaging materials made from polysaccharides, lipids, and proteins has led to the use of natural polysaccharides, which offer an environmentally friendly, cost-effective, and fully biodegradable alternative with promising film-casting capabilities (Bizymis & Tzia, 2022). Despite their advantages, edible films face several drawbacks, including higher production costs, poor barrier properties, and lower tensile strength than traditional plastic films. Nevertheless, they exhibit superior elongation characteristics, making them more flexible than their plastic counterparts. Temperature plays a significant role in edible films' physical and mechanical properties, with material strength typically decreasing when temperatures exceed the glass transition point (Poonia & Mishra, 2022). However, to overcome the drawbacks of edible films used in food coatings, such as poor mechanical strength, limited barrier properties, and moisture sensitivity, researchers are exploring various methods to enhance their performance. One approach is the incorporation of natural biopolymers like chitosan (Apriliyani et al., 2021), starch, and proteins, (Jiang et al., 2024) which improve the film's tensile strength, water resistance, and antimicrobial properties. Cross-linking agents such as glutaraldehyde and natural additives like essential oils and antioxidants are also being used to increase the stability and shelf life of edible coatings (Garavand et al., 2017). Additionally, the use of nanomaterials like nanocellulose or nanoclays has shown promise in enhancing the barrier properties and mechanical strength of these films (Fatyeyeva et al., 2017). Incorporating functional ingredients through techniques like 3D food printing also allows for the creation of customized coatings with enhanced properties tailored to specific food products. These advancements collectively address the limitations of edible films, making them more efficient and sustainable for food preservation (Xie et al., 2023).

Owing to its thermoplastic characteristics, starch obtained from rice, wheat, cassava, corn, and potatoes has been extensively utilized in formulating edible packaging materials. The potential of utilizing various plant-based polymers, such as those derived from banana, okenia, mango, litchi kernel, pinhão, and lotus seeds, is likely broadening the horizons of edible film research (Poonia & Mishra, 2022). For example, Pająk et al. (Pająk et al., 2019) have explored polysaccharides extracted from starch-rich pumpkin fruits, lentils, and quinoa seeds, evaluating their physicochemical, thermal, and mechanical properties. The research revealed that films crafted from these unconventional starch sources showcased enhanced tensile strength and elongation properties, achieving impressive metrics between 8.98 and 13.85 MPa for tensile strength and 3.35 to 4.44 % for elongation. These innovative films displayed remarkable elasticity and preserved the robust attributes typical of solid materials, merging flexibility with structural integrity in a novel manner. Additionally, Behrestaghi et al. (Behrestaghi et al., 2020) found that carboxymethyl cellulose (CMC) films enhanced with *Artemisia sieberi* essential oil showed promising mechanical and physical properties. Such innovations suggest that non-traditional starch-based films can be a viable alternative to synthetic polymer films in sustainable food packaging solutions.

## 5 Application of edible coatings in food packaging

The growing global pollution crisis has heightened awareness of the environmental impact of plastic waste, underscoring the urgent need for sustainable alternatives to traditional plastic packaging. As society increasingly demands safer, natural, and eco-friendly solutions, there has been a significant shift towards developing preservation technologies that safeguard food for human consumption and prioritize environmental sustainability (Al-Moghazy et al., 2021; Azeredo et al., 2022). These solutions must maintain food's sensory and nutritional qualities while reducing harm to the planet. Plant-based polymers have emerged as promising alternatives, enhancing food coating quality and performance. Incorporating plant-derived extracts into food coatings has gained significant attention due to their accessibility, cost-efficiency, and potential to serve as functional additives, improving the overall efficacy of polymer coatings. These green sources provide a natural, sustainable way to enhance food preservation without compromising food safety or contributing to the growing plastic waste problem (Gat et al., 2020).

### 5.1 Fresh vegetables and fruits

Fruits and vegetables are highly perishable living tissues, making them susceptible to significant postharvest quality degradation. Multiple factors, including postharvest handling and processing techniques and environmental conditions such as temperature, humidity, and exposure to sunlight, influence this loss. To address these issues, there is a growing need for advanced technologies that not only boost production and optimize distribution but also minimize quality degradation and extend the shelf life of produce (Luo et al., 2020). After harvest, the primary contributors to the short shelf life of fruits are their elevated respiration rates, the presence of microbial agents, and the loss of moisture. Biodegradable coatings enriched with anti-browning agents, antioxidants, and antimicrobial agents present a promising solution to combat these factors. These coatings can efficiently minimize moisture loss, stop the ripening process, protect from bacteria contamination, and eventually extend the shelf life of fruit (Jemilakshmi et al., 2020).

Research by Sarker and Grift (Sarker & Grift, 2021) has highlighted the potential of *aloe vera* as an effective coating material. *Aloe vera* offers multiple benefits: it possesses antimicrobial properties, prevents oxidation, reduces moisture loss, regulates gas exchange, and helps preserve the color, firmness, and flavor of fruits and vegetables. This makes it an excellent candidate for extending the shelf life of perishable produce. Additionally, other plant-based coatings have yielded similar positive results, with varying effects on different types of fruits and vegetables. These materials are a step towards more sustainable and effective food preservation technologies, offering a natural alternative to conventional methods (Jemilakshmi et al., 2020).

### 5.2 Meat, poultry, and seafood

Meat and fish exhibit high perishability, rendering them particularly vulnerable to rapid degradation when not stored and managed under optimal conditions (Hashemi et al., 2023). Applying edible coatings and films presents a viable method for extending their shelf life. These coatings are primarily formulated from biopolymers, including polysaccharides, proteins, and lipids, which are recognized for their consumption safety and capacity to serve as carriers for active natural compounds. The coating functions as a barrier that effectively limits microbial proliferation, minimizes moisture loss, prevents the accumulation of waste byproducts, and inhibits the oxidation of lipids, proteins, and pigments. Consequently, this approach enhances the sensory acceptability of the product over an extended period (Hashemi et al., 2023).

**Edible coatings derived from plant-based materials have attracted considerable interest due to their availability, cost-efficiency, and inherent bioactive properties. Comprehensive studies have assessed the use of various plant-derived compounds in coatings for meat, poultry, and seafood products. A notable investigation by Khojah (****Khojah,** 2020**) examines the integration of pomegranate peel extract into an edible coating composed of carrageenan and gelatin, highlighting the potential benefits of utilizing fruit waste as a functional ingredient**. Rich in phenolics and tannins, pomegranate peel extract provides potent antioxidant and antimicrobial properties, inhibiting the growth of aerobic bacteria, psychrotrophs, yeasts, molds, and Enterobacteriaceae.

### 5.3 Dairy and bakery products

Yeasts and molds are the most common contaminants in dairy products, leading to undesirable odors, taste issues, and visible defects. These microbial invaders compromise both the sensory qualities and safety of the product. To address this, edible coatings and films—applied through dipping, spraying, or wrapping—offer an effective solution. These coatings, often enriched with antioxidant and antimicrobial agents, create a protective barrier that extends the shelf life of dairy products by preventing microbial growth (Mileriene et al., 2021; Zulewska et al., 2023). The challenge for dairy producers is clear: to meet consumer expectations for high-quality, safe products, there is a pressing need for innovative solutions. By incorporating these advanced coating technologies, dairy businesses can improve the quality and safety of their products, ensuring they remain wholesome and fresh for longer, ultimately benefiting both consumers and producers(Zulewska et al., 2023).

## 6 Technological and commercial challenges

### 6.1 Regulatory and safety aspects

Adopting edible coatings and films made from natural polymers presents a promising solution for enhancing food safety and sustainability. While these natural alternatives are safer and more eco-friendly than synthetic options, they come with regulatory challenges, particularly concerning flavor and color. Natural polymers, although generally safer, can occasionally impart undesirable tastes or affect the appearance of the food, necessitating stringent regulations to ensure that the coatings remain appealing and safe for consumers (Naqash et al., 2022; Poonia & Mishra, 2022). Moreover, including essential oils and other natural antimicrobial and antioxidant additives in these coatings introduces additional complexity. While beneficial for extending shelf life, these additives can alter the taste and texture and even introduce toxicity risks if used in excessive quantities. As the food industry increasingly turns to natural ingredients, further rigorous testing is essential to ensure that these additives meet safety standards consistently. The inherent variability in natural compounds, which often leads to batch-to-batch fluctuations, necessitates enhanced quality control measures to maintain safety and effectiveness across all product batches. Therefore, while the promise of natural coatings is clear, ongoing research and regulatory oversight are crucial to overcoming these challenges and ensuring the widespread acceptance of edible coatings in the food industry (Poonia & Mishra, 2022; Tran et al., 2024; Yadav, Kumar, Upadhyay, Singh, et al., 2022).

### 6.2 Consumer acceptance and market potential

Consumer perception is a critical factor in determining the commercial success of edible coatings. Research has consistently shown that consumer understanding of the benefits of edible coatings directly influences their acceptance. For instance, in Australia, the rejection of coated apples highlighted how unfamiliarity with the concept can lead to consumer resistance. To overcome this challenge, it is essential for manufacturers to effectively communicate the advantages of edible coatings—such as extended freshness, enhanced shelf life, and improved safety—through clear and informative packaging labels (Alizadeh-Sani et al., 2019; Bizymis et al., 2024).

Furthermore, consumers' preference for natural additives over synthetic alternatives is growing, driven by health and safety concerns. This trend makes it even more important for producers to prioritize natural, plant-based ingredients in edible coatings, ensuring they align with consumer expectations (Hassanen et al., 2023). Transparency regarding the materials used, their safety, and the sensory qualities of the coated food can significantly improve consumer confidence. Addressing these factors, the market potential for edible coatings can be expanded while mitigating any negative perceptions or misconceptions surrounding their use (Bucher et al., 2023; Cloete et al., 2022).

### 6.3 Cost-effectiveness and scalability

The commercial viability of edible coatings is significantly influenced by the cost of sourcing high-quality natural ingredients. Extracting these natural compounds can be expensive and challenging to standardize across production batches, which raises concerns about consistency and cost-effectiveness (Costa et al., 2023). While edible coatings offer promising preservation benefits, the application methods, such as spraying, are not always justify the expense, especially for certain food products. Furthermore, plant-based edible coatings often require a combination of active agents and multiple polymers to achieve the desired protective effects, which increases production costs and complicates large-scale manufacturing. Traditional coating techniques, like the dipping method commonly used for tomatoes, can result in uneven thickness, leading to inconsistent performance (Poonia & Mishra, 2022; Souza et al., 2020). This inconsistency poses a significant hurdle in achieving reliable and scalable applications. To overcome these challenges, there is a pressing need for innovative, cost-effective methods that ensure even coating distribution and enhance compatibility between polymeric materials. Such advancements are crucial for making edible coatings a commercially viable and scalable solution for the food industry (Azevedo et al., 2020; Formiga et al., 2022). By refining application techniques and optimizing natural ingredients, the potential for edible coatings to become a widespread, sustainable food preservation method can be realized.

## 7 Future trends and research directions in edible packaging

The global demand for sustainable food packaging solutions has intensified as concerns over environmental pollution, resource depletion, and food waste rise. Edible packaging, a viable alternative to conventional packaging, is increasingly recognized as a critical innovation in the food industry (Okonkwo et al., 2022; Silue & Fawole, 2024). This trend towards more sustainable, eco-friendly, and functional packaging solutions opens new research and development doors. The future of edible packaging lies in enhancing its performance, integrating advanced technologies, and aligning it with consumer preferences and industry needs. This section delves into three significant future trends in edible packaging: sustainable and biodegradable, smart and functional coatings for active packaging, and integration with other food preservation technologies.

### 7.1 Sustainable and biodegradable edible coatings

Sustainability is the cornerstone of the next generation of edible coatings. As plastic pollution continues to wreak havoc on ecosystems, the food packaging industry is under pressure to reduce its environmental impact. Conventional packaging materials, such as plastic and aluminum, are non-biodegradable and contribute to significant carbon emissions during production and disposal. Edible coatings, particularly those made from natural, biodegradable materials like polysaccharides, proteins, and lipids, offer a promising alternative to minimize waste and reduce the reliance on synthetic materials (Fernandes & Miranda, 2022).

Research in sustainable edible coatings focuses on materials that are biodegradable and capable of preserving food quality. The demand for plant-based, renewable resources is driving the development of innovative coating materials. For example, polysaccharide-based coatings derived from cellulose, starch, and chitosan are gaining attention for their ability to form protective barriers that can prevent moisture loss, oxidation, and microbial contamination in food. These biopolymers are biodegradable but also cost-effective and versatile, making them ideal candidates for scalable food packaging solutions (Iñiguez-Moreno et al., 2024; Islam et al., 2024; Sharma, Pathak, et al., 2024).

In addition to polysaccharides, protein-based coatings, such as those derived from soy, casein, and whey, are being explored for their ability to enhance the mechanical strength and barrier properties of edible films. Lipid-based coatings, often derived from plant oils like beeswax or carnauba wax, offer excellent moisture resistance and oxidative stability. The use of these materials is particularly relevant in food preservation, where moisture control and protection from oxygen are critical factors in maintaining product shelf life.

The future of edible coatings lies in developing composite materials that combine the strengths of different biopolymers. These hybrid coatings are expected to outperform individual biopolymers regarding barrier properties, mechanical strength, and environmental impact. For instance, combining chitosan with other biopolymers like gelatin or alginate has shown promise in creating coatings with enhanced film-forming properties and superior food preservation capabilities (Iñiguez-Moreno et al., 2024; Islam et al., 2024). Further research into natural antimicrobial agents, such as essential oils, plant extracts, and probiotics, can also contribute to making edible coatings more effective in preventing spoilage and extending the shelf life of perishable foods.

### 7.2 Smart and functional coatings for active packaging

Smart coatings for food packaging have emerged as innovative solutions to improve food preservation, enhance consumer safety, and extend shelf life. Smart edible coatings are being developed with the ability to respond to environmental stimuli such as temperature, humidity, and gas composition, providing real-time monitoring and control of food storage conditions (Abdel Aziz & Salama, 2021; Adiletta et al., 2021; Alizadeh-Sani et al., 2019).The three primary categories of smart coatings based on their functionalities are active packaging coatings, intelligent packaging coatings, and self-healing coatings.

• Active Packaging Coatings

Active packaging coatings are designed to interact with the food product or its environment to prolong shelf life and preserve quality. These coatings typically contain substances that actively release or absorb certain compounds to prevent spoilage. For example, coatings that release antimicrobial agents, such as essential oils or chitosan, can inhibit microbial growth on food surfaces, reducing the risk of contamination. A study by Popescu et al. (2022) explored chitosan-based edible coatings with added essential oils for their antimicrobial properties, which helped reduce microbial growth on fruits and vegetables, extending their shelf life. Additionally, coatings that absorb oxygen, moisture, or ethylene gas help to maintain the freshness of perishable items by preventing oxidation or moisture loss. One such example is the incorporation of oxygen scavengers in packaging films for meats and seafood to prevent spoilage due to oxidative processes (Akhtar et al., 2015).

• Intelligent Packaging Coatings

Intelligent packaging coatings are designed to provide real-time information about the condition of the food or the packaging environment. These coatings often contain indicators that change color in response to environmental factors such as temperature, pH, or the presence of certain gases (Pereira de Abreu et al., 2012). Such systems enable consumers and manufacturers to monitor the freshness and safety of food products visually. For example, pH-sensitive indicators are used to monitor spoilage in dairy products or meats. In a study by Alamdari et al. (2021a); Alamdari et al. (2021b), pH-sensitive coatings were applied to meat packaging, which changed color to indicate the onset of spoilage. Temperature-sensitive inks are another example, where coatings change color to indicate whether a food item has been exposed to temperature fluctuations that might compromise its safety. Intelligent coatings provide valuable insights into food quality, reducing food waste and enhancing consumer trust.

• Self-Healing Coatings

Self-healing coatings represent a new frontier in food packaging, as they can autonomously repair minor damages to the coating, such as cracks or punctures. This functionality is achieved through the incorporation of microcapsules containing healing agents that are released upon damage (Wang, McClements, et al., 2024). These coatings offer the potential for improved durability and extended shelf life by maintaining the integrity of the protective barrier. For instance, self-healing coatings made with polyurethane and microcapsules containing healing agents have been developed for food packaging applications. Research by Wang, Gao, et al., 2024 Wang, McClements, et al. (2024) and Lai (2023) demonstrated the use of self-healing coatings in food packaging, where microcapsules containing a healing agent were integrated into the coating, enabling the repair of small punctures, thus preventing the entry of microorganisms and preserving food quality. Self-healing coatings can be particularly valuable in the transportation and storage of food products, where packaging is often subjected to mechanical stress.

One of the key innovations in smart edible coatings is the development of sensors that can detect spoilage or contamination. For example, researchers are working on edible coatings that incorporate pH-sensitive dyes, which can change color in response to changes in the acidity of the food (Satorabi et al., 2021). This could provide consumers and food processors with an easy way to assess the freshness of a product without needing to open the package. Similarly, coatings embedded with moisture-sensitive materials can help control humidity levels within the packaging, preventing mold growth or wilting in fruits and vegetables.

• Nanotechnology in coating

Nanotechnology is another powerful tool enabling the development of intelligent and functional coatings. Nanomaterials, such as nanoclays, nanoparticles, and nanofibers, can be incorporated into edible films to enhance their mechanical strength, barrier properties, and antimicrobial activity (Dehghan & Roomiani, 2020). These materials can also improve the uniformity of coatings, making them more effective in preventing food spoilage. Using nano-sensors within edible coatings could allow for real-time tracking of food quality, providing valuable data for consumers and producers. However, along with these advantages come concerns regarding the long-term stability, potential risks associated with migration, sensory effects, and consumer health. Below is a detailed examination of these aspects.

**Long-term Stability and Reliability** One of the primary concerns with nanotechnology in food coatings is the long-term stability and reliability of these materials. While nanomaterials can provide excellent barrier properties (e.g., reducing oxygen and moisture transfer), their performance over time, especially under varying storage conditions, is a critical factor (Onyeaka et al., 2022). For example, certain nanomaterial-based coatings may degrade or lose their effectiveness under light exposure or high humidity, potentially impacting the protection they provide.

**Migration Risk of Nanomaterials** Another significant concern is the potential migration of nanomaterials from coatings into food. The small size and high surface area of nanoparticles increase their potential for migration into the food product, which could pose a health risk (Enescu et al., 2019). For example, nanoparticles of titanium dioxide, commonly used for their whitening properties in food coatings, may migrate into food, leading to possible ingestion. While research is ongoing, regulators such as the European Food Safety Authority (EFSA) are cautious and have imposed strict guidelines on the use of nanomaterials in food packaging to minimize migration risks (European Food Safety Authority, 2011; Poonia & Mishra, 2022).

**Long-term Effects on Sensory Quality** Nanomaterial-based coatings can also influence the sensory qualities of food, such as taste, texture, and appearance. While some coatings, such as those made with nanocellulose, can improve the texture and appearance of food, others may cause changes in taste or mouthfeel. For example, coatings containing certain nanoparticles could affect the flavor profile of fruits or vegetables. The long-term effects of such changes, especially when consumed regularly, are still under study (Onyeaka et al., 2022).

**Consumer Health and Product Quality** The integration of nanotechnology into food coatings offers promising benefits, but concerns remain regarding consumer health and the overall quality of the product. While nanomaterials can help maintain the nutritional and visual qualities of food, the potential health risks associated with their consumption must be thoroughly assessed (Enescu et al., 2019). The long-term health effects, such as the accumulation of nanoparticles in the body, are not yet fully understood, raising questions about their safety. As a result, consumer awareness and regulatory oversight are essential to ensure that food products coated with nanomaterials do not compromise health. In addition, the long-term reliability of these coatings needs to be balanced with their effectiveness and environmental sustainability (Bizymis & Tzia, 2022).

**Consumer Perception and Acceptance** Consumer acceptance of nanotechnology in food coatings is also an important factor. While nanotechnology offers many benefits, there is public skepticism regarding the safety of nanomaterials in food products. Research has shown that consumers are generally cautious about new technologies, especially when it comes to food safety (Poonia & Mishra, 2022).

• Functional edible coatings

Functional edible coatings are also being designed to preserve food and provide additional benefits. For instance, coatings infused with antioxidants, vitamins, and probiotics are being explored to provide health benefits to consumers while extending shelf life (Aguado et al., 2024; Bizymis et al., 2024). Antioxidants, such as polyphenols from plant sources, can help to reduce oxidative damage in foods, preserving flavor and nutritional value. Probiotic-infused coatings could offer an innovative approach to enhance the health benefits of fermented foods by allowing beneficial bacteria to remain viable during packaging and storage. Integrating active and smart features into edible coatings is a key research direction that could revolutionize how we approach food packaging. These coatings are expected to offer more than just protection; they actively contribute to food preservation, quality control, and consumer safety. As research in this area progresses, smart edible coatings will become increasingly sophisticated, providing a dynamic, responsive solution for food packaging (Bizymis et al., 2024). Smart coatings in food packaging—whether active, intelligent, or self-healing—offer innovative solutions to enhance food preservation, safety, and shelf life. Active packaging coatings interact with the food or the environment to improve freshness and reduce spoilage, intelligent packaging coatings provide real-time information on the condition of the food, and self-healing coatings ensure the durability of packaging by repairing damage autonomously. These advancements hold significant promise for the future of sustainable and efficient food packaging.

### 7.3 Integration with other food preservation technologies

The future of edible packaging will focus on the coatings themselves and their integration with other food preservation technologies. By combining edible coatings with techniques like modified atmosphere packaging (MAP), refrigeration, and UV light treatment, it will be possible to create synergistic effects that significantly extend the shelf life of food products (Shahvandari et al., 2021). Combining multiple preservation methods can simultaneously address different aspects of food spoilage, ensuring that foods remain fresh, nutritious, and safe for extended periods. For instance, integrating edible coatings with MAP can enhance their effectiveness by reducing the oxygen content around the food slowing down the growth of aerobic microbes. Edible coatings that are permeable to gases can be designed to work in conjunction with controlled atmospheres, optimizing the internal gas composition to reduce microbial activity and preserve freshness. This combination of technologies could benefit fresh produce, which is highly susceptible to microbial contamination and spoilage (Nekera et al., 2023).

Additionally, edible coatings can be combined with refrigeration and freezing technologies to create a more robust preservation system. The barrier properties of edible coatings can reduce moisture migration during freezing, preventing freezer burn and preserving the texture and quality of frozen foods (Pié-Amill et al., 2024). In conjunction with refrigeration, edible coatings can help maintain the quality of perishable items like meat and dairy by preventing moisture loss and slowing down microbial growth without the need for synthetic preservatives. UV light treatment, shown to reduce microbial contamination on food surfaces, could also be integrated with edible coatings to provide a multilayered approach to food preservation. Coatings can be designed to protect sensitive nutrients and flavors during UV treatment while allowing the antimicrobial properties of UV light to enhance the safety and shelf life of food products (de Menezes et al., 2024; Tang et al., 2025).

Integrating edible coatings with other preservation technologies will enable a more comprehensive approach to food preservation, addressing the complex challenges of microbial contamination, spoilage, and nutrient degradation. This holistic approach will meet the growing demand for longer-lasting, sustainable, and safe food products.

## 8 Conclusion

Edible coatings offer a promising solution to the global challenges of food preservation, waste reduction, and environmental sustainability. By forming protective, consumable barriers, these coatings mitigate spoilage, extend shelf life, and maintain food quality while reducing dependency on plastic packaging and synthetic additives. Their versatility, stemming from biopolymers and natural additives, caters to various applications, from fresh produce to processed meats and bakery products. Innovations in composite and nano-based formulations further enhance their functional properties, ensuring greater efficiency and scalability. However, realizing the full potential of edible coatings requires overcoming obstacles such as high production costs, consumer education, and regulatory constraints. Integrating edible coatings with other advanced preservation technologies presents an opportunity to amplify their benefits, creating synergistic effects that support sustainable food systems. As the food industry shifts towards eco-friendly practices, edible coatings stand out as a pivotal innovation, marrying sustainability with functionality to meet the evolving demands of modern consumers. By fostering continued research and investment, adopting edible coatings can significantly contribute to a greener, healthier future in food packaging and preservation.

## CRediT authorship contribution statement

**Arun Karnwal:** Writing – original draft, Validation, Supervision, Resources, Methodology, Formal analysis, Conceptualization. **Gaurav Kumar:** Writing – review & editing, Validation, Software, Resources, Methodology, Formal analysis. **Rattandeep Singh:** Writing – review & editing, Visualization, Validation, Resources. **Manickam Selvaraj:** Writing – review & editing, Validation, Resources, Project administration, Data curation. **Tabarak Malik:** Writing – review & editing, Resources, Investigation, Formal analysis, Conceptualization. **Abdel Rahman Mohammad Al Tawaha:** Writing – original draft, Resources, Formal analysis, Conceptualization.

## Declaration of competing interest

The authors declare that they have no known competing financial interests or personal relationships that could have appeared to influence the work reported in this paper.

## Data Availability

No data was used for the research described in the article.
